# Metal–Organic Frameworks (MOFs) and Their Composites for Oil/Water Separation

**DOI:** 10.1021/acsomega.4c07911

**Published:** 2024-11-19

**Authors:** Abdullah M. Abudayyeh, Lila A.M. Mahmoud, Valeska P. Ting, Sanjit Nayak

**Affiliations:** † Institute of Condensed Matter and Nanosciences (IMCN), Université catholique de Louvain Louvain-la-Neuve, Walloon Brabant BE 1348, Belgium; ‡ School of Chemistry, University of Bristol, Bristol BS8 1TS, United Kingdom; § Research School of Chemistry & College of Engineering, Computing and Cybernetics, The Australian National University, Canberra ACT 2602, Australia; ∥ Bristol Composite Institute, School of Civil Aerospace and Design Engineering, University of Bristol, Queens Building, Bristol BS8 1TR, United Kingdom

## Abstract

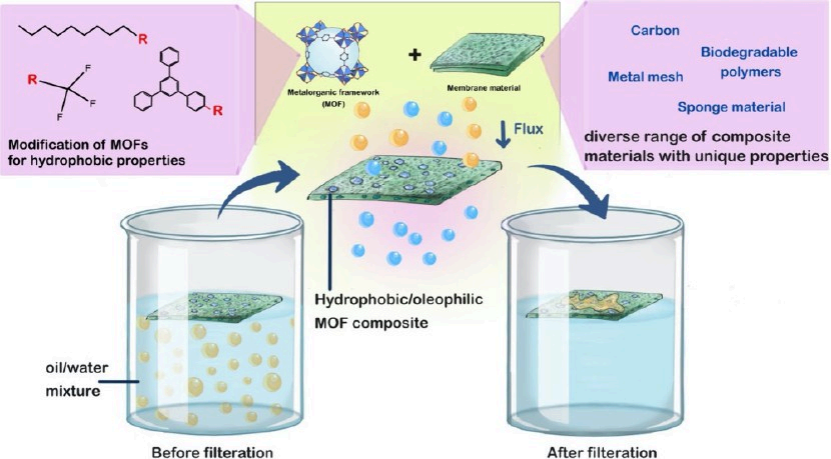

Contamination of water by oil-based pollutants is a major environmental problem because of its harmful impact on human life, marine life, and the environment. As a result, a wide range of materials are being investigated for the effective separation of oil from water. Among these materials, metal–organic frameworks (MOFs) and their composites have emerged as excellent candidates due to their ultraporous structures with high surface areas that can be engineered to achieve high selectivity for one of the phases in an oil/water mixture for efficient water filtration. However, the often nanocrystalline/microcrystalline form of MOFs combined with challenges of processability and poor stability in water has largely limited their use in industrial and environmental applications. Hence, considerable efforts have recently been made to improve the performance and stability of MOFs by introducing hydrophobic functional groups into the organic linkers and fabricating polymer-MOF composites to increase their stability and recyclability. In addition, the use of biobased or biodegradable MOF composites can be particularly useful for applications in natural environments. This Review presents recent advances in the field of hydrophobic MOFs and MOF-based composites studied for the separation of oil from oil/water mixtures, with an account of future challenges in this area.

## 1 Introduction

Water pollution involves the contamination of natural bodies of water such as oceans, lakes, and groundwater with harmful chemicals that are not only detrimental to human health but also have a significant impact on wider environment and ecosystem. Hydrocarbon contamination has been identified as one of the main contributors to water pollution, which can result from food waste, sewage, and industrial effluents. In the mining industry, a standard mining operation can produce up to 140 000 L of oil-contaminated wastewater per day.^1^ Leaking oil pipelines and fracking operations have also been reported to contaminate ground and surface water.^2^ In addition, oil spills from tankers can deposit huge quantities of oil contaminants into the oceans. For example, more than ∼342 000 t of crude oil were spilled into European water in two catastrophic oil spills, namely, the Torrey Canyon in Cornwall (England, 1967) and the Amoco Cadiz in Brittany (France, 1978).^3^ These accidents caused significant damage to ecological systems and marine life, not to mention the depletion of resources and laborious efforts required to eliminate the toxic organic chemicals and other types of pollutants released during the spill. Although strict measures and regulations have been put in place for the transportation of oil, oil spill incidents have increased over the last couple of decades. Investigations into the effect of oil spill contamination on human health suggest a correlation with acute physical, psychological, genotoxic, and endocrine effects.^4^ Consequently, the purification of water from oil residues is a key area of research. Considerable efforts have been made to develop effective and inexpensive materials for water treatment.^5^ Regulations have been imposed by societies and governments to ensure clean water seawater and wastewater through rigorous treatment. Current techniques for oil/water separation to mitigate oil contamination are mainly based on physical treatment methods, which include gravity-assisted separation, centrifugation, filtration, and flotation. Other methods based on electrochemical or biological treatments are also occasionally used. In general, most industrial oil/water separation methods are laborious, energy and capital intensive, and often inefficient due to a low selectivity for oil over water.^6^

Besides the above methods, material-based approaches have also been investigated for oil/water separation. A wide range of materials have been developed and tested for oil/water separation over the last two decades. These include porous carbon-based materials, sponge-based materials, fibrous materials based on biomass,^7,8^ and polymer materials,^9^ as well as extensive work on nanomaterials for oil separation, which can be found elsewhere.^10−12^ The surface of a material to be used in oil/water separation needs to be tuned in such a way as to repel water by having nonpolar components (hydrophobic, or “water-hating”) while bearing high affinity to absorb (for bulk material) or adsorb (for filter materials) oil (oleophilic or superoleophilic, “oil-loving”) to allow the separation of oil from oil/water mixtures.^11^ Materials with opposite affinities can also be used. For example, materials for water removal from oil/water mixtures could have highly polar surfaces, so they display hydrophilic (water-loving) and superoleophobic (oil-hating) characters.^13^ It is widely believed that both the surface topography and the chemical composition of a material determine its superhydrophobicity/superoleophilicity.^14^ Based on that, approaches for tuning the surface of a material intended for oil/water separation either by chemical modification or by assimilation within composites in combination with matrix materials such as sponges, clays, polymers, foams, or carbon materials have recently attracted much interest.^15^ Nevertheless, there is a need to circumvent the fact that some composite materials have a high tendency to absorb water on their own, which seriously limits their performance in oil/water separation.

Metal–organic frameworks (MOFs) are a class of three-dimensional coordination polymers connecting metal clusters (nodes) and organic linkers (struts) through strong coordination bonds, often forming permanent voids.^16^ Due to their high porosity and high surface area along with possible incorporation and opportunity for surface modification by desired functionalities, MOFs have been the subject of great interest for their potential applications in various fields,^16^ like gas storage,^17^ separation,^18^ catalysis,^19^ sensing,^20^ heavy metal removal from water,^21^ herbicides,^22,23^ and drug delivery.^24−26^ Owing to a handy ability to incorporate a diverse range of chemical compositions, thousands of MOFs have been synthesized so far. Among them, more than 100 hydrophobic MOFs,^27^ in addition to their composites, have been synthesized and studied for water treatment and water/oil separation.^6,28,27,29^

It may also be the case that when some rationally designed hydrophobic MOFs are incorporated into highly water repellent surfaces, such as activated resins or fluorinated graphene, the resulting MOF-based composite can have much better hydrophobic behavior,^13,30^ leading to better separation performance for oil/water mixtures.

Due to the poor hydrolytic stability,^31^ and the typically nanocrystalline or powder forms of many MOFs, with no “free standing ability”, direct application of MOFs in oil/water separation might be limited to dispersion, which may result in poor recoverability and recyclability and insufficient absorption capacity in some cases. Building upon that, two main strategies to improve oil/water separation by MOF-based materials have been reported in the literature: the first is post-synthetic modification of the surface of MOFs to improve their hydrophobic/oleophilic properties, and the second is embedding hydrophobic MOFs into other supporting materials to form composites or membranes, for example, by incorporating MOFs into a polymer matrix,^31^ fibers, or sponges^32^ or through the intercalation of MOFs within porous networks.^30^

The following sections will provide an up-to-date account of the advances in hydrophobic MOFs and their composites used for the separation of oil/water mixtures. In the first part, approaches used to synthesize hydrophobic MOFs are briefly discussed, followed by an overview of the metrics used to assess the performance of the MOF composites for oil/water separation. In the following part, the difference in performance between as-prepared hydrophobic MOFs and their composites is critically discussed. Additionally, attempts to synthesize Bio-MOFs and biodegradable MOF composites and their potential application in oil/water separation are also highlighted. Finally, the challenges in this important field of study are outlined, with potential directions for future development.

## 2 Experimental Techniques for Evaluation of Water/Oil Separation

There are some quantitative and qualitative experimental tests to assess the efficiency of materials for water/oil separation. These include tests of hydrophobicity, such as water contact angle (WCA) and absorption capacity measurements, recyclability, and competitive water–hydrocarbon (usually toluene) adsorption experiments. The water content of the MOF composite can also be indicated by IR spectroscopy via monitoring bands originating from OH stretching and H–O–H bending at 3400 and 1621 cm^–1^, respectively. Thermogravimetric (TG) measurement provides not only a quantitative analysis of the amount of water adsorbed by the MOFs/MOF composites but also the stability of the framework during MOF–water interaction within a broad temperature range.^29^ A number of parameters are used to evaluate the performance of MOF composites for the removal of oil from oil/water mixtures, such as water contact angle, absorption capacity, separation flux, separation efficiency, and durability. These parameters will be discussed in more detail in the following sections. The formulas used in the following sections are based on normal pressure, where liquid flow is caused by gravity and no external pressure is used.

### 2.1 Water Contact Angle

**Figure 1:**
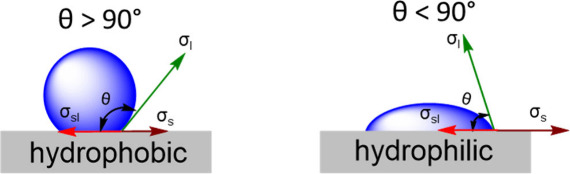
Water contact angle of (left) a hydrophobic surface (θ < 90°) and (right) a hydrophilic surface θ > 90°. Figure was adopted and modified based on ref (27).

The hydrophobic behavior of MOFs and their composites is usually indicated by the water contact angle (WCA) measurement. MOF composites with WCA angles greater than 90° (Figure 1, left) suggest that the surface repels water and has low wettability. In such instances, the MOF composite is regarded as hydrophobic. In contrast, if the surface has affinity to water, the WCA will be less than 90° (Figure 1, right) and the MOF composite is considered as hydrophilic. Rarely, but of great interest, when the MOF or its composite has a contact angle with water greater than 150°, the material is considered as superhydrophobic.

The contact angle of a water drop (θ) on a surface can be calculated using Young’s equation (eq 1).
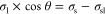
1In this equation, σ_l_ represents the surface tension of water, θ is the contact angle between the water drop and the surface, σ_s_ is the surface free energy, and σ_sl_ is the interfacial tension between the surface and the water drop.^27,29^

### 2.2 Absorption cCapacity

Another parameter to assess the performance of a MOF composite for oil/water separation is its absorption capacity (*k*). It measures the ability of MOF composites to absorb oil, expressed as grams per gram of sample, by measuring the mass of the MOF composite sample before and after immersion in an oil/water mixture. Equation 2 is used to calculate the absorption capacity of different materials.^33^
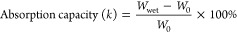
2In this equation, the amount of oil absorbed by the MOF composite, which can be calculated by subtracting the mass of the MOF composite before absorption (*W*_0_) from the mass of the MOF composite after absorption (*W*_wet_), is compared to its initial mass (*W*_0_). MOF composites reported to date have shown a wide range of absorption capacities between 5 and 600 wt % (Table 1).^30^

**Table 1:**
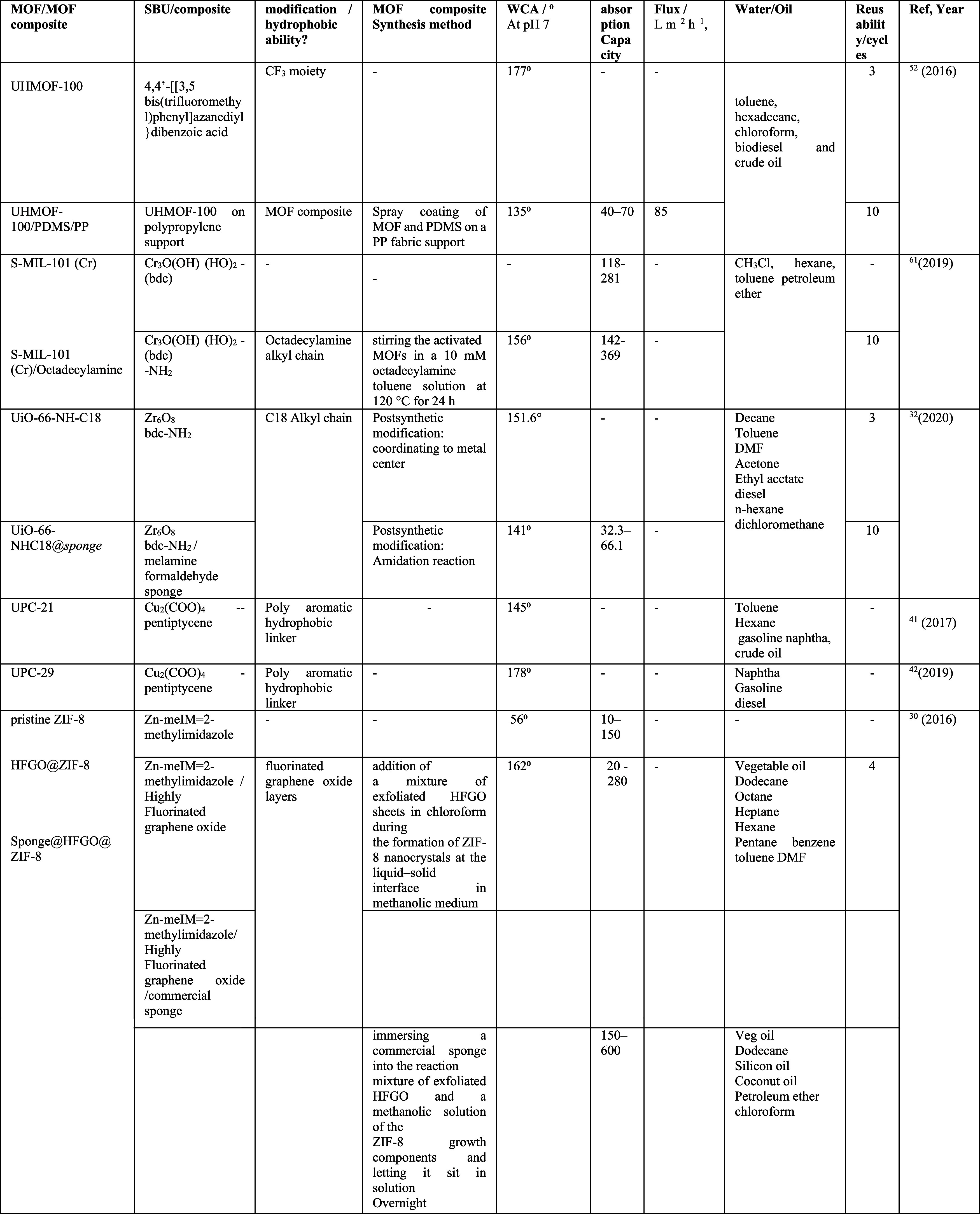

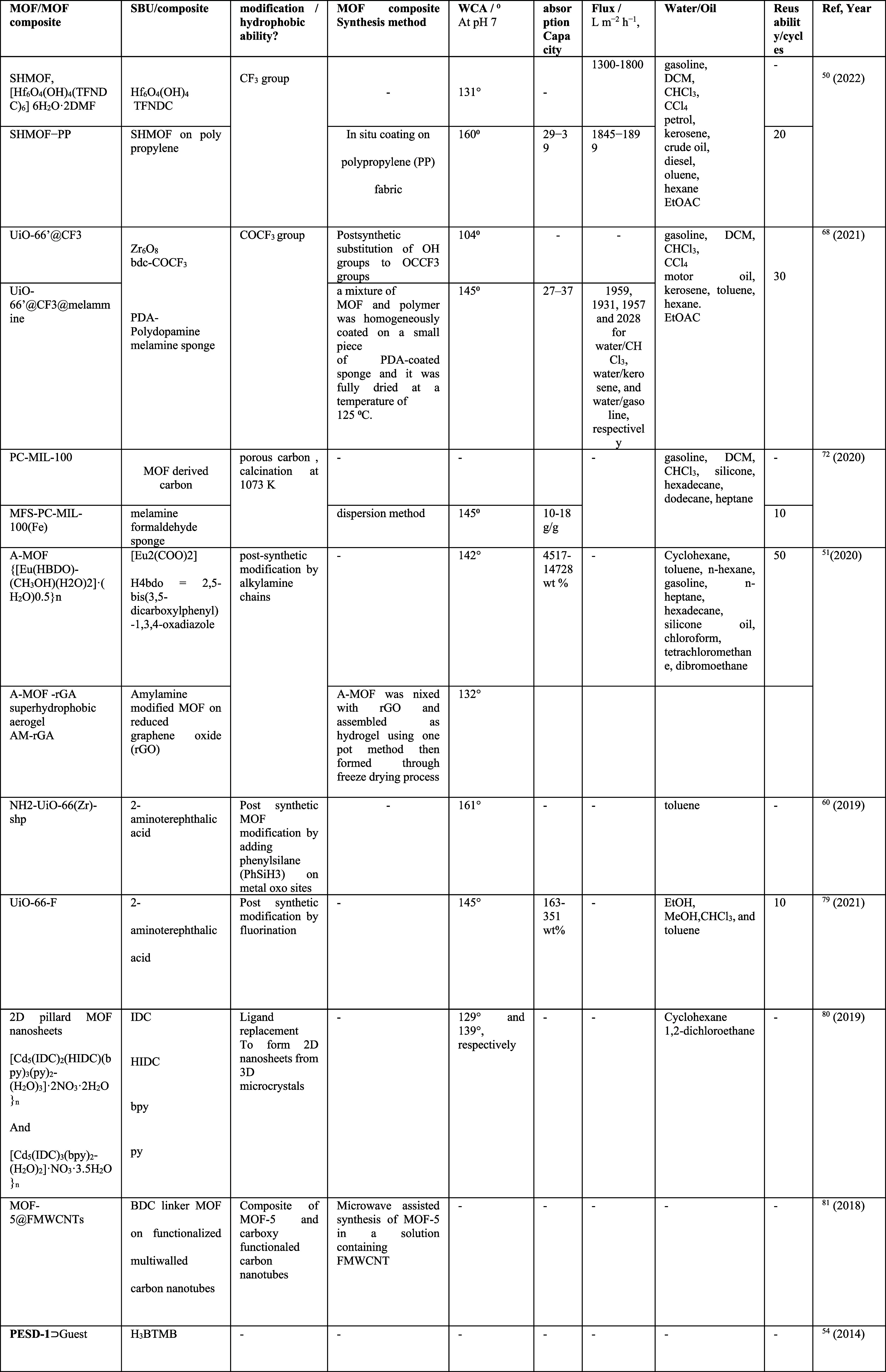

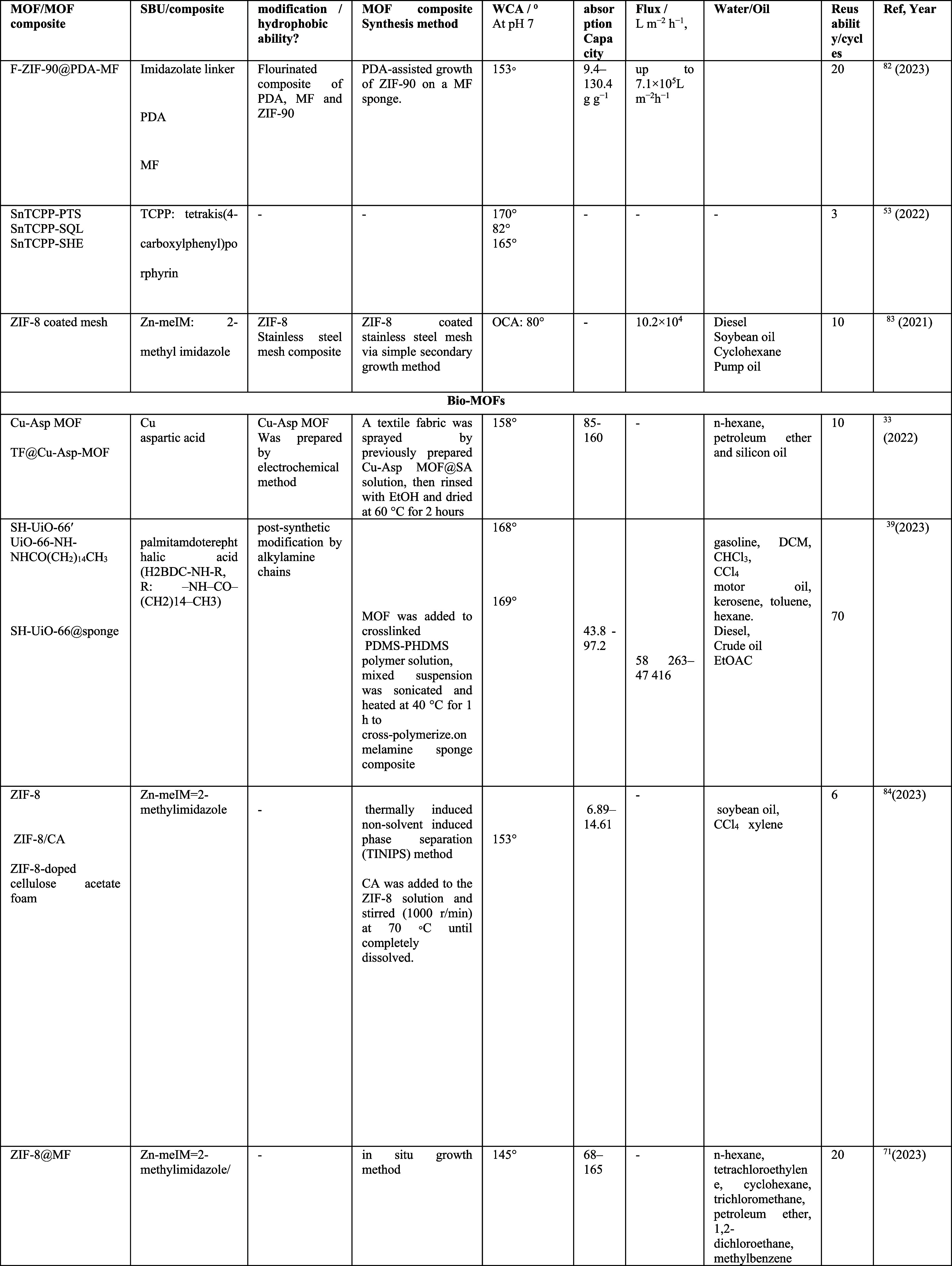

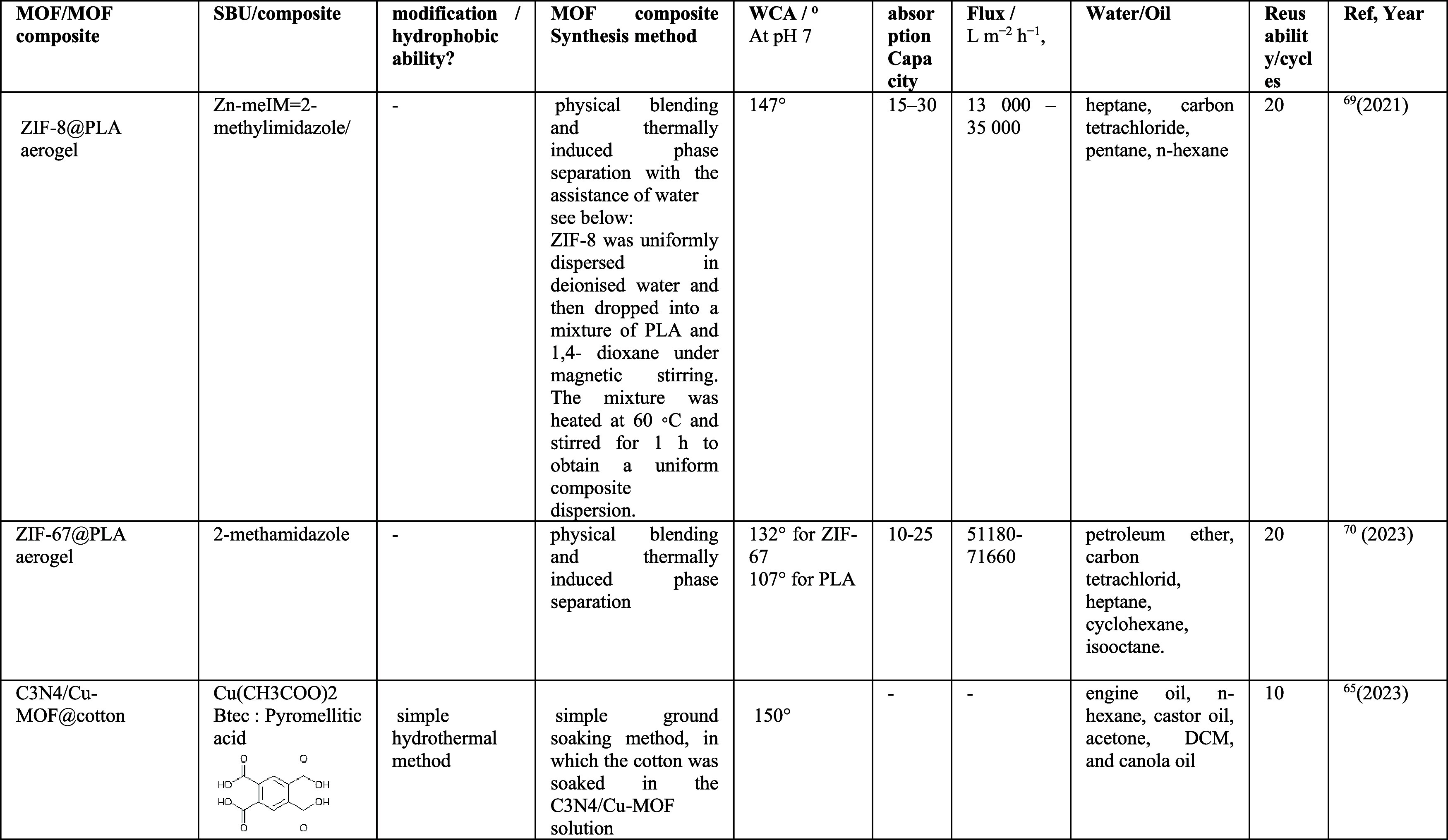
Summary of Hydrophobic MOFs/Composites with Their Hydrophobic Properties, Water Contact Angle (WCA), and Applications in Oil/Water Separation^a^^79,80,81,82,83,84^

a shp: superhydrophobic, BTFM: 2,5-bis(trifluoromethyl)terephthalic acid, DABCO: 1,4-diazabicyclo[2.2.2]octane, PDMS: polydimethylsiloxane, PP: polypropylene, HFGO: highly fluorinated graphene oxide, TFNDC = 1-(2,2,2-trifluoroacetamido)naphthalene-3,7-dicarboxylate, MFS: melamine formamide sponge, H_4_bdo: 2,5-bis(3,5-dicarboxylphenyl)-1,3,4-oxadiazole, BTFM: 2,5-bis(trifluoromethyl)terephthalic acid, DABCO:1,4-diazabicyclo[2.2.2]octane, H3IDC: imidazole-4,5-dicarboxylic acid, bpy: 4,4′-bipyridine, py: pyridine, BDC: 1,4-benzenedicarboxylate, MWCNT: multi-walled carbon nanotubes, FMWCNT: functionalized multi-walled carbon nanotubes, MF: melamine formaldehyde.

### 2.3 Separation Flux

Separation flux reflects the amount (by volume) of a liquid mixture (*V*) passing through per unit of effective area (*A*) of a MOF composite per unit time (*t*).^33^Equation 3 below is used to calculate the separation flux of a MOF composite material during the separation of oil/water mixtures. High flux through a membrane and good retention of oil are crucial factors for membranes for oil/water separation technology.^34,35^ This means that an efficient membrane must allow a high amount of the oil/water mixture to be filtered through in a given period of time while maintaining high retention of oil. Flux is dependent on many factors, including intrinsic factors of the membrane such as surface roughness, surface chemistry, pore size, and thickness.^36^
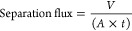
3

### 2.4 Separation Efficiency

Separation efficiency (*R*) is a measure of the membrane’s ability to separate oil from an oil/water. mixture. *R* is an important metric and can be used to measure the efficiency of a MOF or MOF composite for the separation of oil from an oil/water mixture. Equation 4 is usually used to calculate *R*.
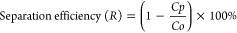
4Here, *Co* is the initial oil concentration in the oil/water mixture and *Cp* is the final concentration of oil in the oil/water mixture after separation.^33^ The separation efficiency depends on the wettability of the membrane, as it reflects the membrane’s ability to separate the oil and water phases by their surface energy differences.^37^ Degradation of the materials and other factors like fouling can eventually reduce the porosity of membranes and can negatively impact the separation efficiency of the material.

### 2.5 Durability and Recyclability

Durability of the MOF material used in oil separation can be defined as the ability to withstand harsh conditions without being chemically altered or physically damaged during separation. One way to test the durability of the MOF composite for oil separation from water is to determine the number of cycles that MOF composites can perform oil/water separation processes without significant degradation. Also of note, harsh conditions can have a great impact on the performance and hence the durability of the MOF composite; those include but are not limited to exposure to high or low pH, variation in temperature and pressure, or any sort of mechanical forces that cause damage. Durability is highly dependent on the composition and mechanical properties of the material such as toughness, hardness, and tensile strength. Many MOFs are prone to degradation at low or high pH, and sometimes they are incorporated within composite materials to improve their chemical and mechanical stability.

The reported recyclability of the MOF composites used in oil/water separation ranges between 6 cycles for ZIF-8-doped cellulose acetate foam^38^ and up to 70 cycles for a superhydrophobic melamine sponge composite (SH-UiO-66@sponge) of a Zr-based MOF with a long-chain hydrocarbon-based linker.^39^ Different approaches have been used to study the recyclability of the composites, including reuse upon simple gravity filtration through the membranes and continuous operation using peristaltic pumps.^38,39^

## 3 Hydrophobic MOFs for Oil/Water Separation

Hydrophobicity is often an indicator of the oleophilicity of a material,; therefore, it gives an idea of the expected performance for oil/water separation. By definition, if the MOF material displays WCA > 90° (see Figure 1), then the MOF is considered hydrophobic; if the WCA ≥ 150°, then it suggests that the MOF has superhydrophobic character.^29^ A number of parameters contribute toward the hydrophobicity of MOFs, including the presence of hydrophobic linkers or the attachment of hydrophobic groups on the linker or on coordinatively unsaturated metals by post-synthetic modification. Crystal engineering also plays an important role in aligning hydrophobic groups on the surface of the MOFs, enhancing hydrophobic properties of the MOFs. Each of these parameters is discussed in the following section.

### 3.1 Use of Hydrophobic Linkers

**Figure 2:**
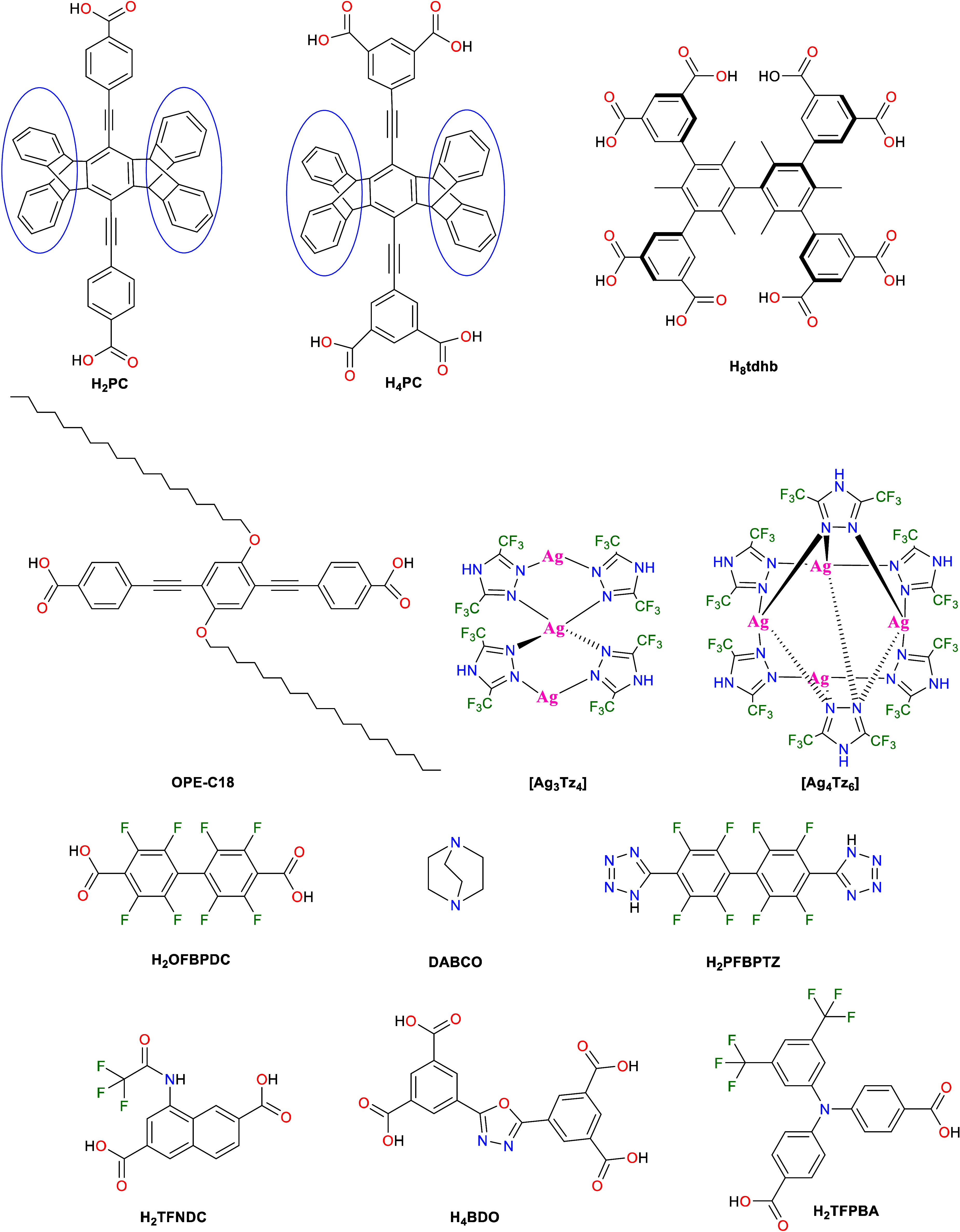
Selected building blocks used to synthesize hydrophobic MOFs. H_2_PC (cyclopentiptycene dicarboxylic acid ligand),^40^ H_4_PC (cyclopentiptycene tetracarboxylic acid ligand),^46^ H_8_tdhb (3,3′,5,5′-tetrakis(3,5-dicarboxyphenyl)-2,2′,4,4′,6,6′-hexamethylbiphenyl,^43^ OPE-C18 (dialkoxyoctadecyl-oligo-(*p*-phenyleneethynylene)dicarboxylate,^47^ Tz (3,5-bis(trifluoromethyl)-1,2,4-triazolate),^48^ H_2_OFBPDC (2,2′,3,3′,5,5′,6,6′-octafluorobiphenyl-4,4′-dicarboxylic acid),^49^ H_2_TFNDC (1-(2,2,2-trifluoroacetamido)-naphthalene-3,7-dicarboxylic acid),^50^ H_4_BDO (2,5-bis(3,5-dicarboxylphenyl)-1,3,4-oxadiazole),^51^ and H_2_TFPBA (4,4′-([3,5-bis(trifluoromethyl)phenyl]azanediyl)benzoic acid).^52^

The hydrophobicity of the linkers can originate from several factors, including the presence of π-electron-rich aromatic groups, long alkyl chains, or fluorinated groups. The use of π-bond-rich hydrophobic linkers such as cyclopentacytene^40−42^ or aromatic linkers^43^ is one of the primary strategies for the synthesis of hydrophobic MOFs. Pentiptycene is a type of iptycene, which is a rigid organic ligand with multiple phenyl rings bonded to a bicyclooctatriene unit system. The rigidity of the phenylbicyclooctatriene head unit does not allow iptycene ligands to pack efficiently in the solid state, resulting in inevitable void spaces being formed by the structure. Thus, iptycene ligands have attracted much interest in material and supramolecular chemistry.^44,45^ In 2015, MacLachlan et al. first reported a cyclopentiptycene dicarboxylic acid ligand, H_2_PC (Figure 21), to synthesize a paddle-wheel zinc-based MOF (PMOF).^40^ Subsequently, in 2017, Sun and co-workers demonstrated how using the cyclopentiptycene tetracarboxylic acid analogue,^46^ H_4_PC (Figure 2), can tune the hydrophobicity and water stability of a copper-based MOF, UPC-21, with excellent potential for application in oil/water separation.^41^ Following the same strategy, in 2019, Zhang and co-workers synthesized another copper-based water-resistant MOF, UPC-29, but this time using the ditopic H_2_PC ligand (Figure 2) with high hydrophobic character.^42^ Another example where aromatic phenyl-rich hydrophobic linkers have been used to prepare hydrophobic MOFs was reported in 2017 by Chen et al., who used the octatopic linker H_8_TDHB (3,3′,5,5′-tetrakis(3,5-dicarboxyphenyl)-2,2′,4,4′,6,6′-hexamethylbiphenyl; Figure 2).^43^ The authors reported a copper-paddlewheel MOF (BUT-155) composed of H_8_TDHB.^43^ Unlike previous copper-paddlewheel-based MOFs, such as HKUST-1 (also known as MOF-199), BUT-155 showed high stability in water, retaining its structure even after treatment in boiling water for 10 days. Furthermore, the authors highlighted the potential of this MOF to extract aniline vapor in preference to other organic and water vapors, which was attributed to its acidic property due to open metal sites in its structure and high hydrophobicity, respectively.^43^

He et al. have reported an organotin-based MOF (SnTCPP-SHE) with superhydrophobicity.^53^ The Sn-based MOFs were synthesized solvothermally with various Sn-based ligands and a TCPP linker and have demonstrated a WCA of up to 170° due to the hydrophobic nature of organotin building units. Additionally, the SnTCPP-SHE MOF has shown a surface area of 3940 m^2^ g^–1^, which is among the highest BET surface areas reported to date for hydrophobic MOFs.

**Figure 3:**
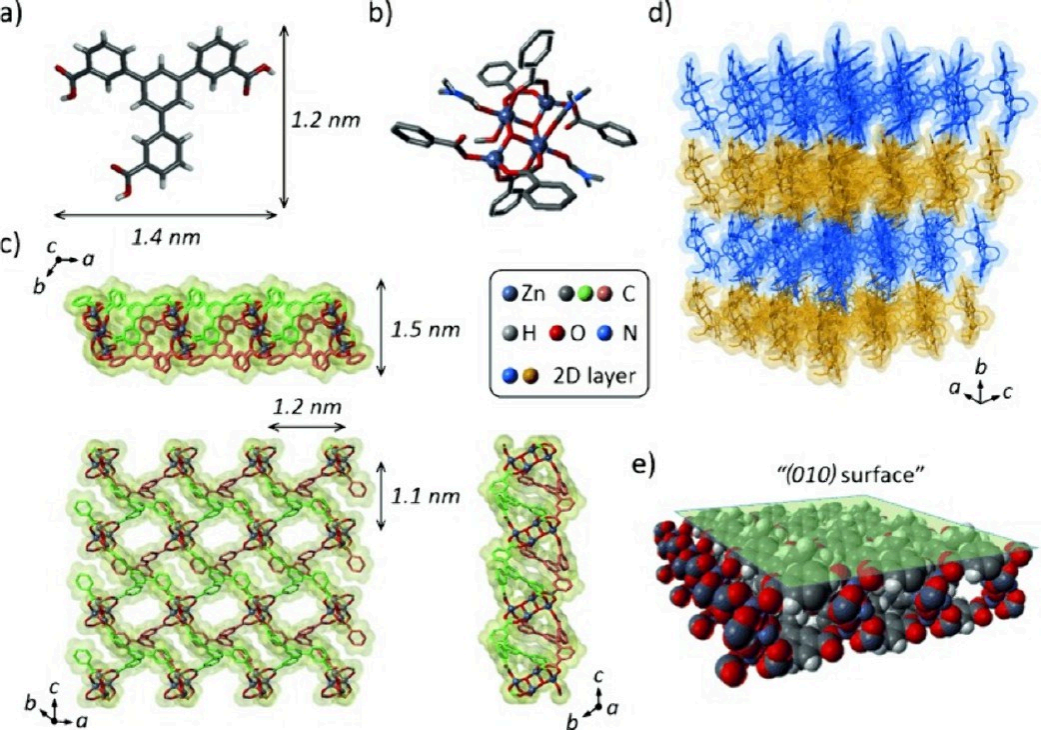
Molecular structures showing (a) the H_3_BTMB linker and (b) the coordination mode of the PCP’s [Zn_4_(μ_3_-OH)_2_]^6+^ cluster. (c) 2D layer arrangement of the coordination polymer, (d) stacking of 2D layers to form 3D structures, and (e) the (0*k*0) surface with terminating phenyl moieties. Adapted with permission from ref (54).

In another study, Kitagawa et al. reported a superhydrophobic porous coordination polymer (PCP) with high roughness and low-energy surfaces.^54^ The coordination polymer was formed by reacting Zn(NO_3_)_2_·4H_2_O with the organic linker 1,3,5-tris(3-carboxyphenyl)benzene (H_3_BTMB). As is shown in Figure 3, the crystalline nature of the PCP results in terminal phenyl moieties that create “microtextures”, resulting in a WCA of >150°. The strategically selected low-symmetry linker resulted in a large area of the crystal surface being covered by the phenyl rings, which contributed to the hydrophobic properties of the MOF.

As shown in the above studies, the choice of the linker greatly influences the hydrophobicity of the resulting MOFs. Interestingly, in 2016 researchers synthesized a novel MOF with switchable hydrophobic and hydrophilic behavior.^55^ The Zn-based MOF was assembled from a 1,4-benzenedicarboxylate and carborane-based linker. With the enhanced hydrophobicity of the carborane linker, the MOF powder exhibited a WCA of 140°.

In 2017, Sun and co-workers^41^ prepared a Cu-paddle-wheel MOF, UPC-21, using the pentiptycene tetracarboxylic acid as a hydrophobic linker. UPC-21 showed excellent hydrophobicity with a water contact angle of 145°. Additionally, UPC-21 exhibited excellent separation efficiency (99%) for various oil/water mixtures such as toluene/water, hexane/water, gasoline/water, and naphtha/water. However, a lower efficiency was observed for the crude oil/water mixture, which was attributed to its high viscosity and multicomponent nature. In 2019, Zhang and co-workers^42^ reported the synthesis of another Cu-paddle-wheel-based MOF, UPC-29, in which the authors used a pentiptycene ditopic linker instead of the tetratopic linker used in UPC-21, resulting in UPC-29 with a high water contact angle of 178° indicating excellent hydrophobic behavior, though no oil adsorption capacity has been reported for this material.

Another strategy to build hydrophobic MOFs is to use organic linkers bearing long alkyl chains. In 2016, Maji and co-workers were reported the use of a dialkoxyoctadecyl-oligo-(*p*-phenyleneethynylene)dicarboxylate (OPE-C18) linker (Figure 2) to prepare a Zn-based three-dimensional hydrophobic MOF (NMOF-1).^47^ Interestingly, the octadecyl alkyl chain (C18) present in this MOF protruded outward from the 1D chain of Zn-OPE-C18. Structural characterization of this MOF also revealed that π–π stacking between the chains allowed the formation of a 2D structure, whereas the 3D structure was built out via interactions between alkyl chains. This led to the formation of NMOF-1 with reduced surface free energy and a WCA of 160–162°, indicative of the superhydrophobicity of this MOF. Furthermore, NMOF-1 showed self-cleaning behavior with retained superhydrophobicity over a pH range of 1–9 in a concentrated ionic medium.^47^

**Figure 4:**
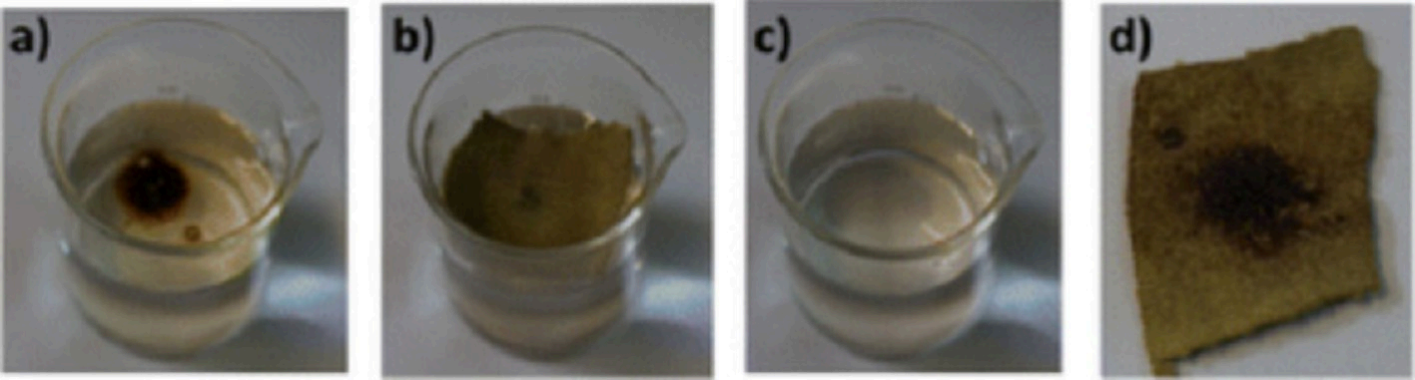
(a) Sample of crude oil/water mixture. (b) HEX-MOF@cotton composite applied on the surface of the mixture. (c) Successful removal of crude oil. (d) HEX-MOF@cotton fabric after oil sorption. Adapted with permission from ref (56)

Another strategy to induce hydrophobicity is to integrate long-chain alkyl groups as part of the linkers. For example, in a study by Manos et al. the use of long-chain alkyl-functionalized amino terephthalic acid linkers for Zr-based MOFs was investigated.^56^ The aminoterephthalic acid linker was reacted with various alkylaldehydes to form alkyl-functionalized amine derivatives, which were then used to synthesize Zr-based MOFs. The derived MOFs showed an unusual Zr(IV) 6-c net as a result of steric hindrance caused by the alkyl groups. The MOFs have shown hydrophobic properties, with WCAs ranging from 150° to 154°, except for the hexyl-amino-H_2_BDC MOFs, which showed wettability to water. The MOFs and their composites with cotton fabrics showed efficient separation of crude oil from water in both static and continuous flow modes (Figure 4).

**Figure 5:**
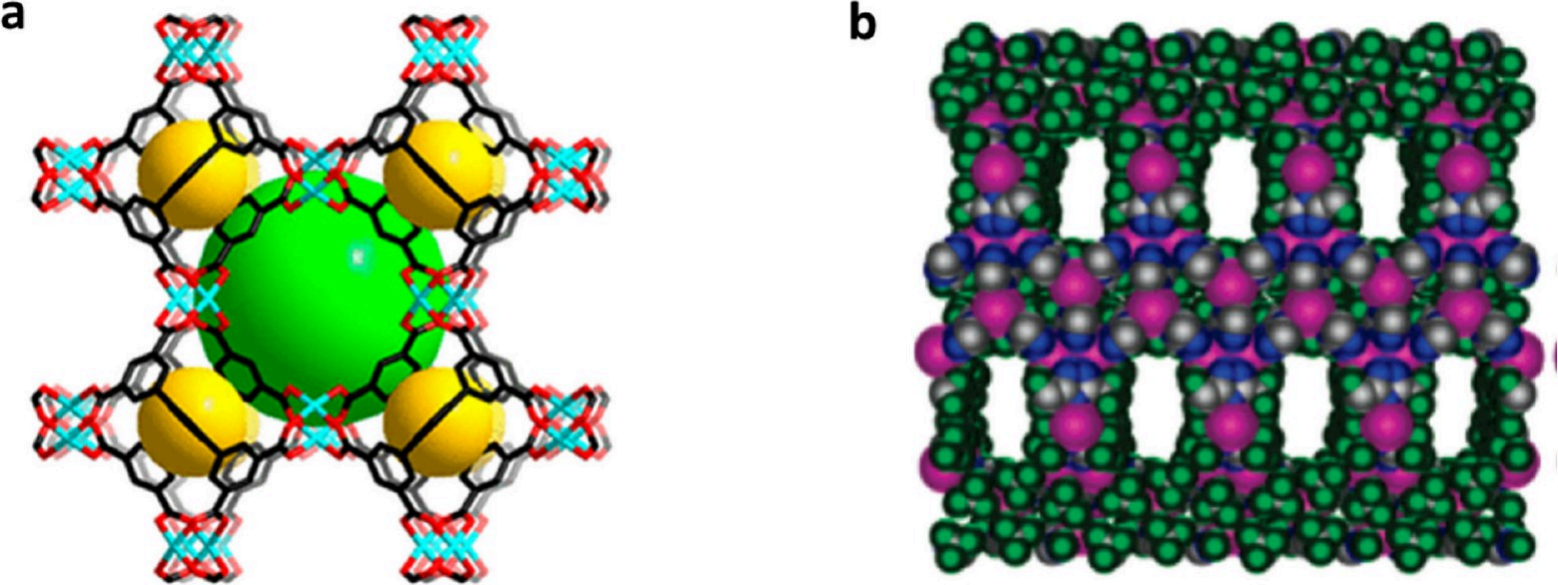
Framework structure of (a) Cu-paddle-wheel-dhb^8–^ MOF (BUT-155) (adapted with permission from ref (43)) and (b) fluorous MOF (FMOF-1) (dapted with permission from ref (48)).

Another class of hydrophobic MOFs is fluorine-functionalized MOFs (F-MOFs). In this class of MOFs, fluorine is integrated into the MOF either by incorporating it within the inorganic metal cluster of the MOF or through the use of fluorine-rich organic linkers. Fluorine-functionalized organic molecules are often hydrophobic and are stable against oxidation, making them highly sought after due to their stability.^57^ Early reports of F-MOFs can go back to 2007 when Omary and co-workers utilized the perfluorinated 3,5-bis(trifluoromethyl)-1,2,4-triazolate ligand (Tz, Figure 2) to synthesize a class of Ag(I)-based fluorine-functionalized MOFs (FMOF-1 and FMOF-2).^48,58^ These highly fluorinated MOFs contain a tubular channel with [Ag_4_Tz_6_] clusters (Figure 5). The authors have reported that FMOF-1 and FMOF-2 displayed high adsorption toward various liquid hydrocarbons, such as *n*-hexane, cyclohexane, benzene, toluene, and *p*-xylene, in preference to water, with FMOF-2 showing a toluene adsorption capacity double that of FMOF-1 due to its larger pore size.^48^

**Figure 6:**
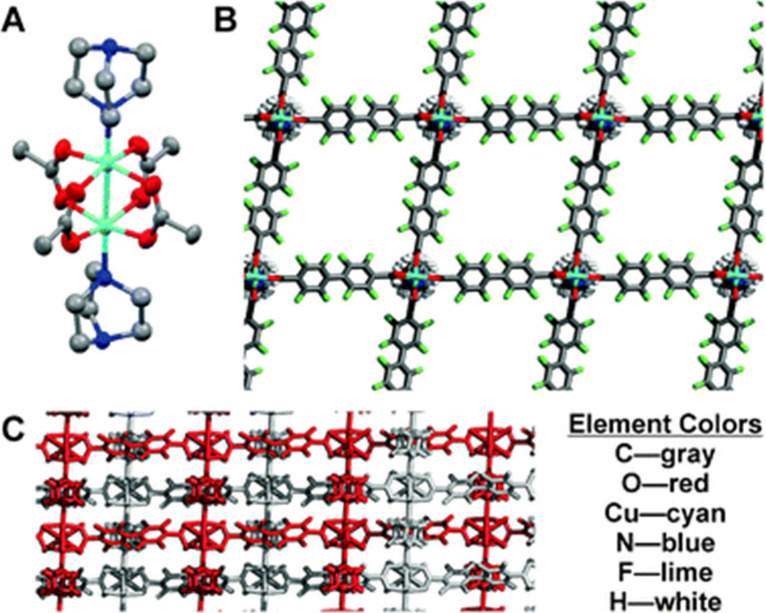
X-ray crystal structure of MOFF-2. (A) Secondary building unit of MOFF-2. (B) View along the one-dimensional channels in MOFF-2. (C) Side-on view of MOFF-2 with two independent nets shown in different colors. Adapted with permission from ref (49).

In 2013, Miljanić et al.^49^ used the perfluorinated aromatic linkers F1–F3 (Figure 2) to synthesize copper-based hydrophobic MOFs (MOFF-1, MOFF-2, and MOFF-3). MOFF-1 was prepared by combining the 2,2′,3,3′,5,5′,6,6′-octafluorobiphenyl-4,4′-dicarboxylic acid (H_2_OFBPDC) linker with Cu(NO_3_)_2_ in a solvothermal reaction, producing 2D methanol-capped layers of Cu_2_OFBPDC. While MOFF-2 was made of the same dicarboxylic ligand (H_2_OFBPDC), diazabicyclo[2.2.2]octane (DABCO) was used as a pillar to interconnect the 2D Cu_2_OFBPDC layers, forming a three-dimensional network (Figure 6). Similarly, the tetrazole-based linker H_2_PFBPTZ (Figure 2) was used to synthesize MOFF-3, bearing a one-dimensional hydrophobic channel in its structure. Contact angle measurements revealed that MOFF-1, MOFF-2, and MOFF-3 are all hydrophobic with WCA = 108 ± 2°, 151 ± 1°, and 134 ± 2°, respectively. In contrast to MOFF-1 and MOFF-3, MOFF-2 crystallizes with no water molecules in its pore voids, another indication of its superhydrophobic behavior.^49^

### 3.2 Post-Synthetic Modification of MOFs for Improved Hydrophobicity

**Figure 7:**
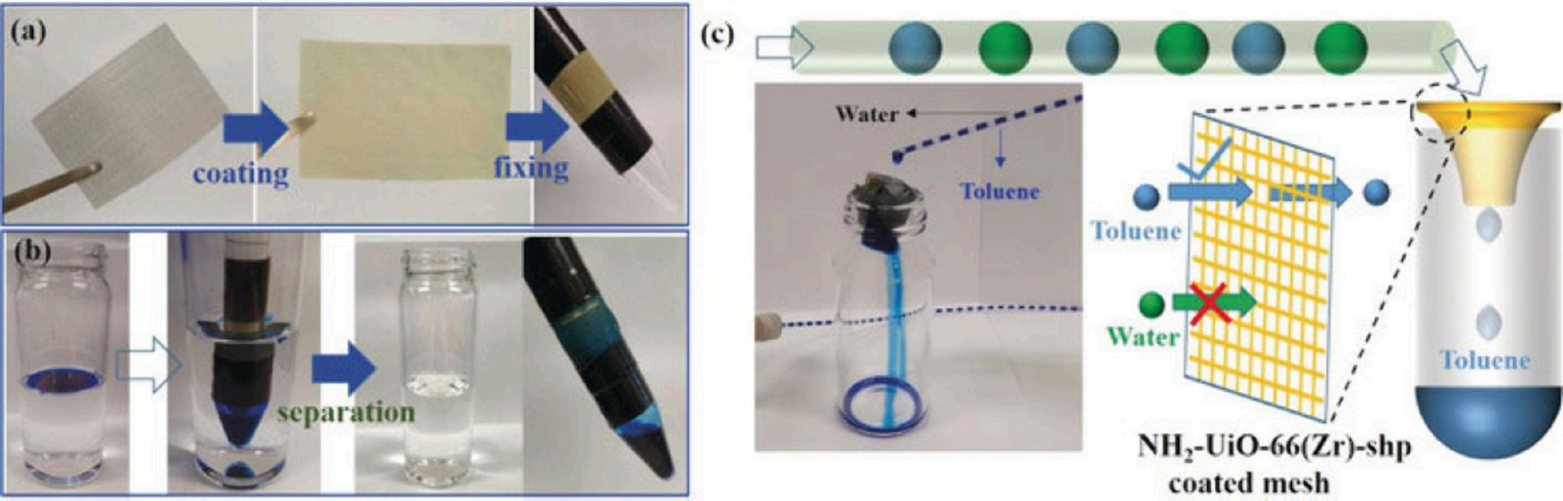
(a) Preparation of a separator by assembling superhydrophobic MOF (NH_2_-UiO-66(Zr)-shp) coated stainless mesh with a test tube collector and (b) selective separation of Oil Blue N labeled toluene from water. (c) Schematic illustration and photograph of the continuous separation of toluene from alternating droplets of water/toluene. Reproduced with permission from ref (60).

Post-synthetic modification (PSM) is another strategy widely used to introduce hydrophobicity in MOFs. PSM involves modifying the structure of MOFs via alteration in the metal centers or linkers by treating the MOFs with suitable reactants. For example, long alkyl chains, aromatic rings, or fluorinated groups can be added by post-synthetic treatment of MOFs to introduce hydrophobicity in the structure. This can be achieved either by (i) coordinating an unsaturated metal site (open metal site) with a ligand containing hydrophobic functional groups or terminal long alkyl chains or (ii) chemical transformation/derivatization of the functional groups in the organic linker, such as functional transformation of an amino group (−NH_2_) into an amide group (R–CO–NH_2_), where R is a long alkyl chain typically with 4–12 carbon atoms. The main advantage of PSM is that it allows for the diverse functionalization of the MOFs without interfering in their core structure as opposed to optimization of the structure of the MOFs during synthesis, which is often more challenging.^59^ An example of oxo-metal cluster decoration can be found in a study done by Sun et al.^60^ In this study the oxo cluster of UiO-66-NH_2_ was post-synthetically modified via grafting of a hydrophobic phenylsilane (PhSiH_3_) by reacting it with the Zr–OH groups. This addition of a hydrophobic phenyl group resulted in the formation of a superhydrophobic MOF system with a WCA of 161°. The phenylsilane-modified MOFs showed excellent resistance to degradation against acid and basic environments compared to UiO-66-NH_2_. To investigate the efficiency of the MOF for separation, the MOF was deposited on a stainless-steel mesh to form a separation filter (Figure 7).

**Figure 8:**
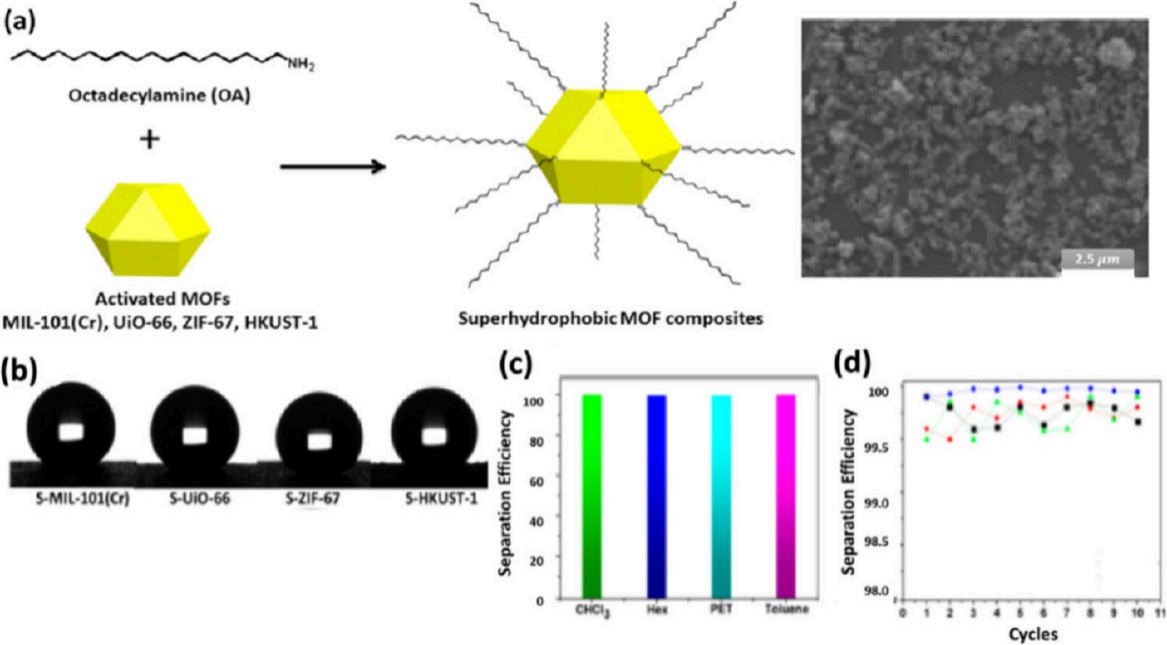
(a) Schematic illustration of the formation of surface-modified superhydrophobic MOF composites using the octyadecylamine amine coordinating ligand. The SEM image shown on the right presents the surface modified S-MIL-101(Cr). (b) Water contact angles of surface-modified MOF composites. (c and d) Separation efficiency of S-MIL-101(Cr) composites for separating oil form different kinds of water–oil mixtures (green, CHCl_3_; blue, hexane; cyan, PET; and red, toluene). Adapted with permission from ref (61).

Another example of utilizing the vacant uncoordinated metal sites for PSM was reported in 2019 by Han and co-workers,^61^ who attached octadecylamine (OA) with the open coordination sites present in the activated MIL-101(Cr), UiO-66, ZIF-67, and HKUST-1 to form superhydrophobic MOFs (S-MOFs) (Figure 8). The prepared MOFs retained their crystallinity and morphology as confirmed by PXRD and SEM. Furthermore, S-MIL-101 (Cr) displayed enhanced superhydrophobic properties with a relatively high-water contact angle (156°) and less water absorptivity. The surface-modified S-MIL-101 (Cr) MOF exhibited separation capacities of 118–281 and 142–369 wt % for the pristine MOF and its functionalized counterpart, respectively.

In addition to modifying the uncoordinated metal sites of the MOF structure, the ligand can also be post-synthetically modified by addition of hydrophobic functional groups. It was shown that amine-functionalized linkers are susceptible to post-synthetic modification and often used to introduce hydrophobic properties. Nguyen et al. have post-synthetically modified the isoreticular series of IRMOFs and MIL-53(Al)^62^ through the addition of medium to long alkyl chains. The hydrolytic stability of the MOFs was assessed by exposure to ambient air and submersion in water for up to 4 days. The added hydrophobicity not only increased the WCA but also helped to stabilize the MOF against degradation, which was evidenced by comparing the PXRD patterns of the samples. Based on the same MOF, Xue et al. grew flower-like MOF-53-OH on a polyacrylonitrile/polyethylenimine (PAN/PEI) membrane to enable the separation of oil from an oil/water emulsion as well as the removal of soluble dyes.^63^ The introduced PAN layer allowed the organic linker in MOF-53-OH to cross-link on the membrane surface, making it superhydrophilic with a high uptake capacity.

Lui et al. developed a superhydrophobic ZIF-90 by performing post-synthetic modification on the pristine ZIF-90 to develop a reusable adsorbent of bioalcohol from water mixtures.^64^ Functionalization of ZIF-90 with pentafluorobenzylamine was performed through an amine condensation reaction in which the pentafluorobenzylamine was reacted with the aldehyde group of the ZIF-90 imidazolate-2-carboxyaldehyde linker. The fluorinated ZIF-9 showed a WCA of 152.4° compared to that of 93.9° for nonfunctionalized ZIF-90. The improved hydrophobicity was attributed to the incorporation of the fluorinated pentafluorobenzylamine within the MOF structure introduced by PSM. Additionally, the MOFs were tested for their hydrolytic and thermal stability by boiling them in water for 24 h. The MOFs showed high chemical and thermal stability. As evidenced by thermal gravimetric analysis (TGA), both the fluorinated and as-prepared ZIF-90 showed a similar plateau region in the temperature range of 80–300 °C without significant weight loss, even after the measurement of water stability.

## 4 MOF Composites for Oil/Water Separation

**Figure 9:**
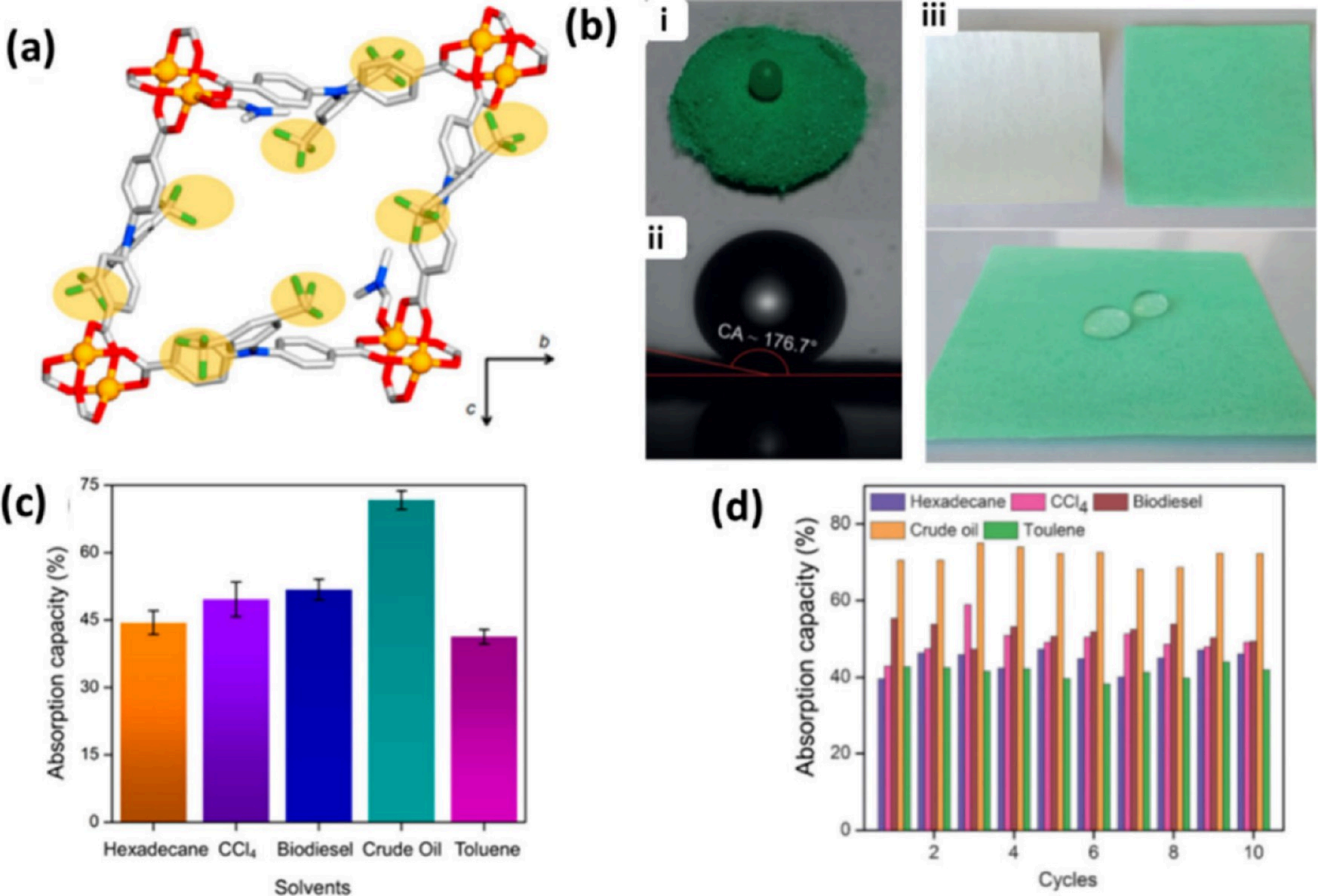
(a) View of single fluorous pores of UHMOF-100 with CF_3_ groups highlighted in yellow. (b) (i) Photographs of a water droplet suspended on UHMOF-100, (ii) water contact angle of UHMOF-100, and (iii) photographs of the membrane base (white) and MOF-coated membrane (green) and (bottom) retention of hydrophobicity for the MOF-coated membrane. (c) Absorption capacities and (d) recycling tests of UHMOF-100/PDMS/PP. Adapted with permission from ref (52)

While many studies have been focused on the development of intrinsically hydrophobic MOFs for the separation of oil and water, there is a growing interest in the development of MOF composites for the enhancement of their properties and extending their applicability. It also takes advantage of integrating desirable properties of the MOFs and the other components (such as the polymer) of the composites. MOF composite membrane materials include mixed matrix membranes, where the MOF acts as a filler in a continuous polymer matrix; polycrystalline membranes, in which a thin crystalline layer of a microporous MOF is deposited on a substrate material for mechanical support; and nanosheet membranes, derived from the fabrication of thin MOF membranes by the layer-by-layer deposition method. Other popular composite materials include incorporating MOFs into structures bearing high surface areas and large pores such as highly fluorinated graphene oxide (HFGO)^30,51^ or environmentally friendly materials, such as cotton,^65^ cellulose,^38,66^ carbon sponges,^67^ biodegradable melamine,^39,68^ and polylactic acid (PLA)^69,70^ Considerable efforts have recently been made to improve the recyclability and environmental compatibility of the MOF–polymer composites. For example, MOFs have been incorporated into biodegradable matrices, such as PLA and other polymers (see below), to improve the stability and environmental compatibility of the MOFs and simultaneously improve the oil separation performance of the polymer using the properties of the incorporated MOF, such as hydrophobicity, surface area, and tunable porosity. In 2016, Ghosh and co-worker reported the “first” ultrahydrophobic Cu-based MOF, [(Cu_4_L_4_(DMF)_4_)(DMF)_3_]_*n*_ (UHMOF-100) (Figure 9), using 4,4′-([3,5-bis(trifluoromethyl)phenyl]azanediyl) benzoic acid (H_2_L = H_2_TFPBA, Figure 2) as a linker with fluorine-rich CF_3_ groups.^52^ The excellent water-repellent and high oil-absorptivity characteristics of this MOF were evidenced by the very high water contact angle (176°) and low oil contact angle (0°). The MOF was spray-coated on a reusable polypropylene support using polydimethylsiloxane (PDMS) as a binding agent to design a membrane for water/oil separation. However, compared to the pristine MOF, the resulting composite UHMOF-100-PDMS-PP showed a lower WCA (135°), which was attributed to the PDMS with its lower WCA of 109°. Generally, the membrane retained a high level of hydrophobicity and oleophilicity, with good separation capacity for various kinds of oil/water mixtures for toluene, hexadecane, chloroform, biodiesel, and crude oil/water mixtures ranging between 40 (for a toluene/water mixture) and 75 wt % (for crude oil/water mixture).

Another strategy for introducing hydrophobicity into the composite is using a hydrophobic substrate as a support for MOFs. Fisher and co-workers used this strategy and reported the development of a hydrophobic/oleophilic zeolite imidazole framework (ZIFs) composite in 2016.^30^ The group reported the synthesis of superhydrophobic/superoleophilic monocrystalline ZIF-8 composites (HFGO@ZIF-8) prepared using a highly fluorinated graphene oxide substrate. The oxygen functionality of the graphene surface allowed both selective nucleation and control over ZIF-8 crystal growth, resulting in the intercalation of the ZIF-8 network between HFGO layers. The resulting composite displayed a high WCA of 162° compared to the WCAs of 56° and 125° for the pristine ZIF-8 and HFGO, respectively. The composite showed a low contact angle of 0° for oil, indicating its superoleophilic nature. The relative absorption capacity for the pristine ZIF-8 was reported between 10 and 150 wt % for different types of oils and organic solvents, whereas the HFGO@ZIF-8 composite showed enhanced capacity with a range of 20–280 wt %. The group investigated further and fabricated a sponge@HFGO@ZIF-8 composite, in which a commercial sponge was immersed overnight in the reaction mixture containing all components of the HFGO@ZIF-8 composite. The resulting sponge@HFGO@ZIF-8 hybrid composite achieved 150–600 wt % oil absorption capacity for various mixtures, including vegetable oil, heptane, DMF, and chloroform. Interestingly, the sponge readily floats on water, permitting scalable practical application of this material.

More recently, ZIF-8 was also incorporated into a melamine formaldehyde (MF) composite for oil/water separation.^71^ The ZIF-8@MF composite was produced by an in situ deposition method. Despite the hydrophilicity of the MF substrate (WCA 54°), the ZIF-8@MF composite displayed high WCA (145°) and very low oil CA of 0°, indicating the superhydrophobicity and superoleophilicity of the resulting composite. Furthermore, the ZIF-8@MF composite achieved adsorption capacities in the range of 68–165 wt % for various oil/water mixtures, with a separation efficiency of around 100% for up to 20 cycles.

Due to their attractive biodegradability and recyclability, poly(lactic acids) (PLAs) have found applications in a variety of composite materials. However, PLA matrices still have limited oil adsorption capacity and poor mechanical properties that limit their wide application in oil/water separation. Due to its high hydrophobicity and superoleophilicity (oil CA 0°), ZIF-8 was also incorporated into biodegradable PLA by solvent-assisted dispersion using sonication and mixing, followed by freeze-drying.^69^ In 2021, Li et al. combined ZIF-8 nanoparticles with a PLA matrix using physical mixing to produce a ZIF-8@PLA composite. The pore size, surface roughness, and surface area of the resulting ZIF-8@PLA composite were successfully tuned by changing the ratio of ZIF-8 in the PLA polymer matrix. In addition, the resulting ZIF-8@PLA composite exhibited improved oil separation performance and significantly improved mechanical properties compared with those of the virgin PLA polymer.

The resulting composite ZIF-8@PLA showed a WCA of 145° and successfully separated the mixtures of oil/water, heptane/water, carbon tetrachloride/water, and pentane/water with a separation capacity range of 15–30 wt %, a flux range of 13000–35000 L m^2–^ h^–1^, and an efficiency of around 100% for up to 20 cycles.

**Figure 10:**
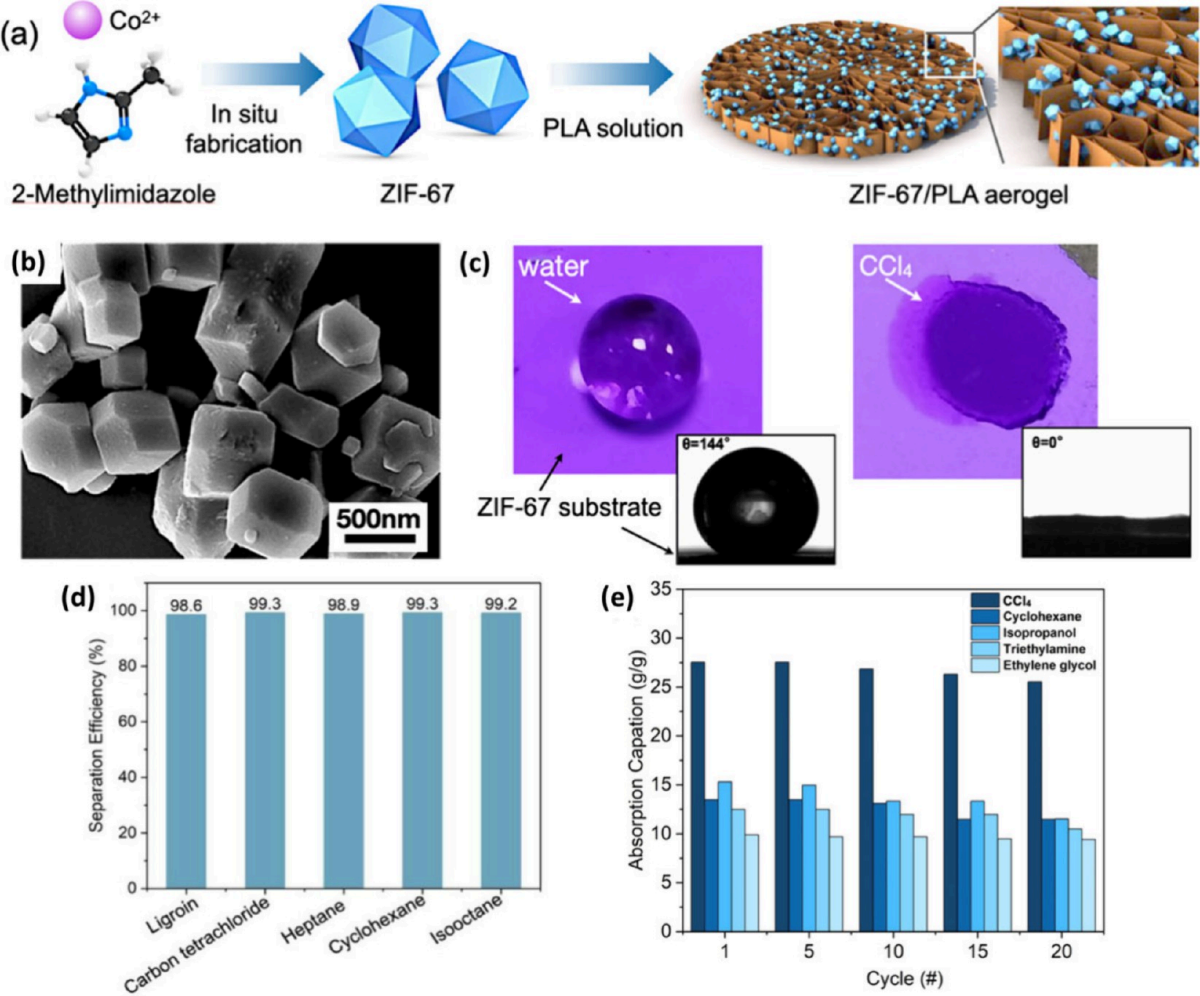
(a) Schematic showing the preparation of the ZIF-67@PLA aerogel/composite with a honeycomb structure. (b) SEM image of ZIF-67, (c) digital images with corresponding CA measurement of the ZIF-8 substrate with a drop of water (144°) and a drop of CCl_4_ (0°), (d) separation efficiency, and (e) adsorption capacity with cycle test of the ZIF-67@PLA aerogel/composite for different oil/water mixtures. Adapted with permission from ref (70).

In another study using a similar method, PLA was formulated with ZIF-67 to produce a honeycomb ZIF-67@PLA composite for oil/water separation (Figure 10).^70^ A typical optimized loading of 3 wt % ZIF-67 in PLA produced the ZIF-67@PLA composite with the highest wettability, displaying a WCA of 132° compared to that of 107° for the PLA on its own. The composite showed a moderate absorption capacity (10–25 wt %) with a good flux range (51180–71660 L m^2–^ h^–1^) and excellent separation efficiency (∼98–100%), and usability was tested for 20 cycles for petroleum ether/water, carbon tetrachloride/water, heptane/water, cyclohexane/water, and isooctane/water mixtures.

**Figure 11:**
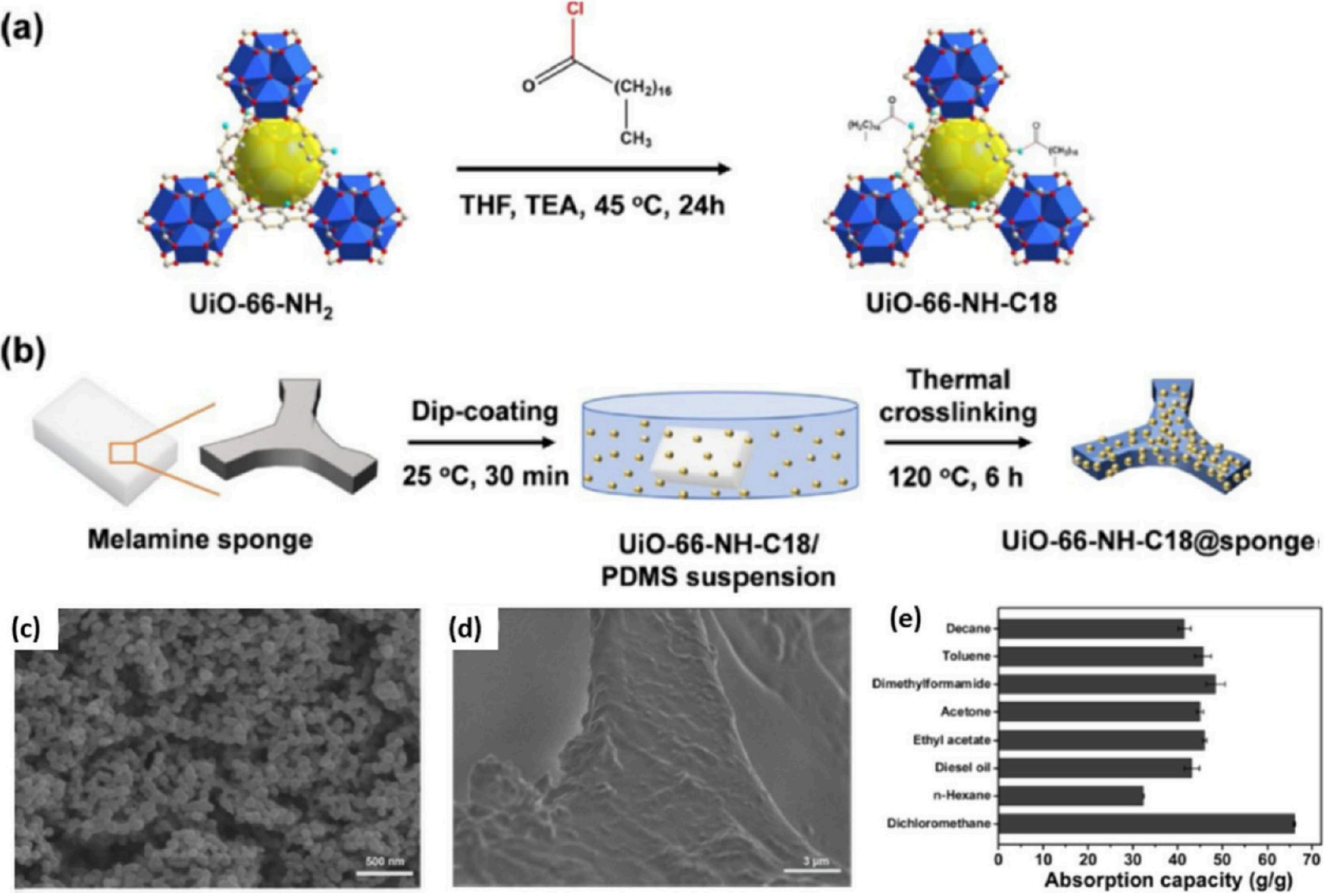
Schematic showing the preparation of (a) superhydrophobic UiO-66-NH-C18 and (b) its composite UiO-66-NH-C18@sponge. (c, d) SEM images of UiO-66-NH-C18 and its composite UiO-66-NH-C18@sponge. (e) Absorption capacity bar chart of the UiO-66-NH-C18@sponge composite for various kinds of organic/water mixtures. Adapted with permission from ref (32).

Huang and co-workers prepared a hydrophobic MOF, UiO-66-NH-C18, by amidation of the amino group of UiO-66-NH_2_ with octadecanoyl chloride (Figure 11).^32^ Owing to the long alkyl chain covalently attached to the surface of the MOF, the resulting UiO-66-NH-C18 showed superhydrophobic properties with a high WCA of 152°. UiO-66-NH-C18 adsorbs much less water than the pristine UiO-66-NH_2_ (28% of that for UiO-66-NH_2_). Furthermore, the group prepared a superhydrophobic composite, UiO-66-NH-C18@sponge, by dip-coating a melamine formaldehyde sponge with UiO-66-NH-C18. This composite was found to display an enhanced absorption capacity ranging between 32.3 and 66.1 wt % for various oils and organic solvents.

**Figure 12:**
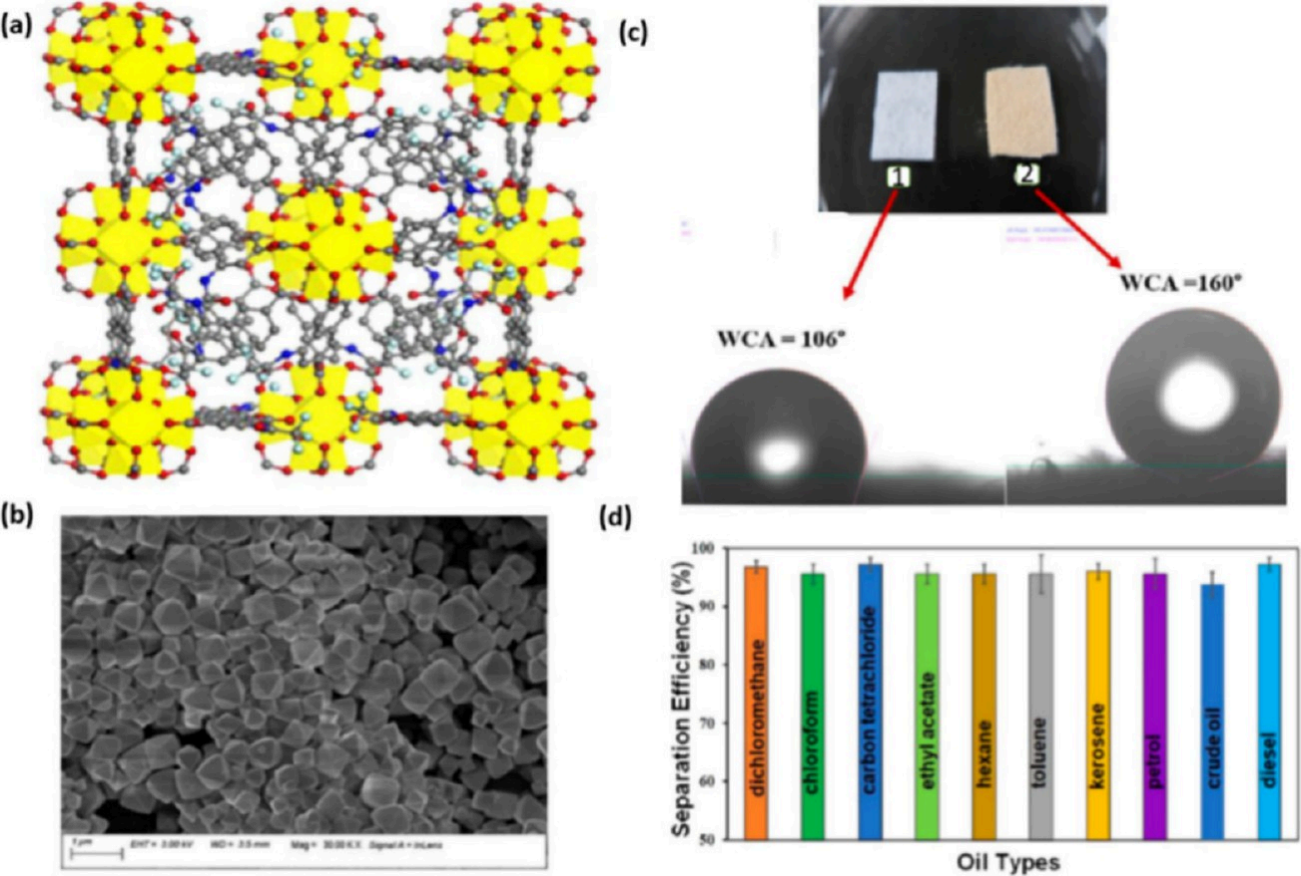
(a) Simulated crystal structure of SHMOF (Zr polyhedron; C, O, N, and F are displayed in yellow, gray, red, and blue, respectively). (b) SEM image of SHMOF. (c) WCA value of (1) native polypropylene (PP) fabric (106°) and (2) SHMOF–PP composite (166°) with increased hydrophobicity due to the immobilization of SHMOF onto the PP fabric. (d) Separation efficiency (%) of the SHMOF–PP composite for various oil/water mixtures. Reproduced with permission from ref (50).

Biswas et al.^50^ synthesized a hydrophobic Hf-based MOF (SHMOF). In situ coating of SHMOF on polypropylene (PP) fabric produced a superhydrophobic SHMOF@PP fabric composite (Figure 12) with a WCA of 160°, high separation efficiency (93–99%), good recyclability (up to 20 cycles), and moderate absorption capacity (29–39 wt %) for separating crude oil from an oil/water mixture. The same group synthesized a hydrophobic Zr-based MOF (1′@CF_3_) through post-synthetic modification of the free hydroxyl groups in the UiO-66-OH (1′) by attaching OCCF_3_ groups.^68^ The stability of the MOFs was tested by stirring them at room temperature for 24 h in water acquired from various sources (river, pond, tap, and seawater) at a broad pH range of 2–14. PXRD patterns of the treated samples were compared with those of the pristine MOFs, and their identical patterns indicated excellent stability. The resulting hydrophobic MOF was utilized in the preparation of a robust melamine-based composite (1′@CF_3_@melammine) using polymethylhydrosiloxane (PMHS) to help anchor the prepared MOF on the melamine sponge. The resulting 1′@CF_3_@melammine composite showed a WCA of 145° compared to that of 104° for the polymeric material alone. The absorption capacity and separation efficiency were reported as 27–37 wt % and 95–99% for the MOF alone (1′@CF_3_) and the composite (1′@CF3@melammine), respectively. Furthermore, 1′@CF_3_@melammine was tested successfully for separation of the oil/water mixture for up to 50 cycles.

Additionally, The same group used a nonfluorous long-carbon linker, palmitamodoterphthalic acid, to prepare a superhydrophobic MOF, SH-UiO-66.^39^ SH-UiO-66 was deposited on a melamine-based sponge using the PDMS–PHDMS cross-linking agent. The resulting composite SH-UiO-66@sponge showed almost the same WCA as the pristine MOF (168°), indicating the superhydrophobic nature of both the MOF and the composite. The SH-UiO-66@sponge composite displayed a good oil/water separation efficiency range between 43.8 and 97.2 wt % and a flux range of 58263–47416 L m^2–^ h^–1^ for various oil/water mixtures (see Table 1).

The mesoporous iron-based MOF MIL-100 was transformed into its porous carbonaceous form, PC-MIL-100(Fe), by Cabello and co-workers.^72^ Incorporation of PC-MIL-100(Fe) into a commercially available melamine-formaldehyde sponge (MFS) resulted in a highly hydrophobic and oleophilic composite sponge MFS-PC-MIL-100(Fe). The composite showed a WCA of 145° and 0° oil contact angle. The absorption capacity of the MFS-PC-MIL-100(Fe) composite for various oils was in the range of 10–17%, showing stability up to 17 cycles. It is worth noting that this composite was fabricated by the simple dispersion of PC-MIL-100(Fe) into melamine-formaldehyde sponges.

**Figure 13:**
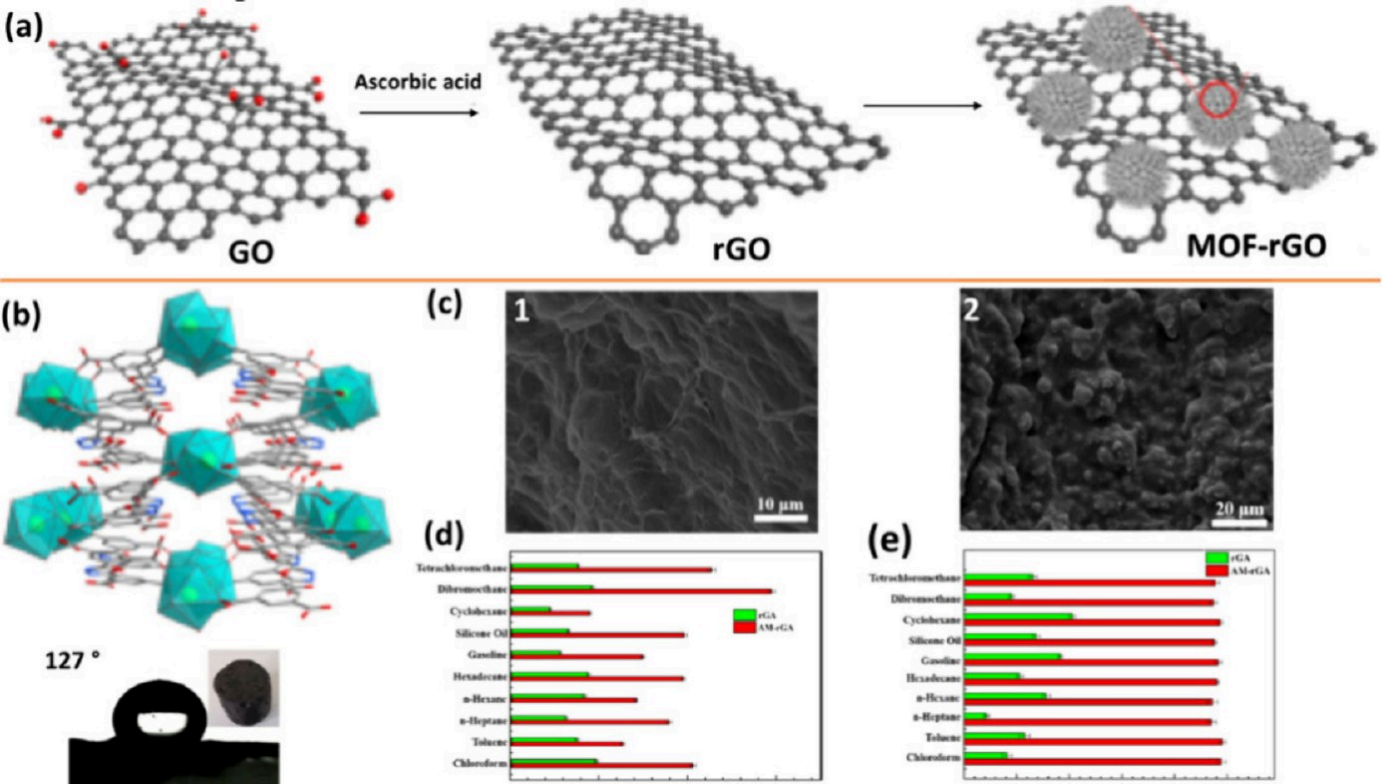
(a) Proposed scheme for the self-assembly process of rGO and amylamine-modified (Eu-bdo-COOH) MOF microspheres (one is highlighted with a red circle). (b) 3D framework of Eu-bdo-COOH MOF with the water contact angle (127°). (c) SEM images of (1) rGO and (2) MOF-rGO. (d) Dbsorption capacities and (e) separation efficiencies of MOF-rGA and rGA toward 10 different types of oil/water mixtures. Reproduced with permission from ref (51)

Control of crystal morphology combined with post-synthetic modification has also been used to control the hydrophobicity of MOFs, followed by their integration into composites. According to a report by Fan and Yang et al., adjusting the crystallization process changes the morphology of the Eu-bdo-COOH MOF from hexagons to microspheres, resulting in enhanced hydrophobicity.^51^ The resulting MOF, Eu-bdo-COOH (Figure 13), displayed high stability in a broad pH range and at high temperatures up to 400 °C. In order to modify the hydrophobicity of the resulting MOF, free “hydrophilic” carboxylic acid groups (WCA = 32°) were transformed into “hydrophobic” alkyl groups through post-synthetic modification of the Eu-bdo-COOH MOF via reaction with a series of alkylamines (ethylamine to octylamine) to produce amyl-MOFs (A-MOFs) displaying a water contact angle of 142°, indicating a high degree of hydrophobicity for the modified MOF. The group combined the hydrophobic amyl-MOFs with reduced graphene oxide (rGO) to produce an A-MOF-rGA composite for oil/water separation. The A-MOF-rGA composite displayed a somewhat reduced WCA angle (127°) due to the very low hydrophobicity of the rGO within the composite, exceptional absorption efficiency for various oil/water mixtures, including an impressive absorption capacity of 14 728 wt % for a dichloromethane/water mixture, and 100% separation efficiency without any decay in performance after 50 cycles.

**Figure 14:**
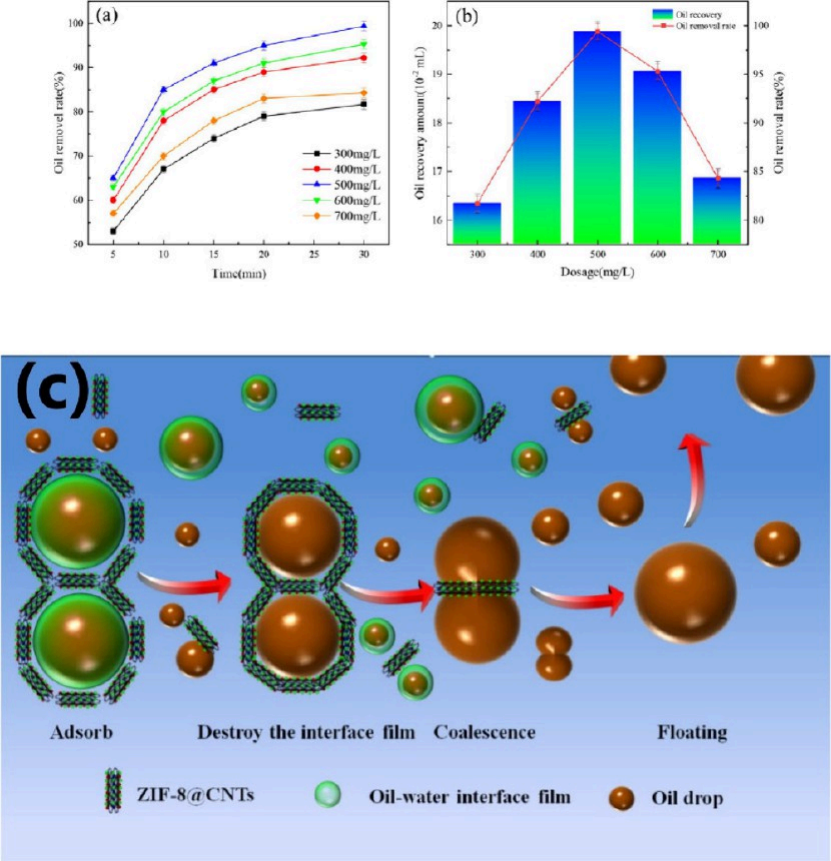
(a) Removal rate (%) of oil over a period of 30 min and (b) amount of oil recovery over dosage ranging from 300 to 700 mg/L. (c) The demulsifying mechanism of ZIF-8@CNT. Reproduced with permission from ref (73).

Hydrophobic composites can take advantage of the hydrophobicity of one or more of the components of a composite. In a study by Zhang et al., ZIF-8 was loaded on top of oxidized carbon nanotubes (CNTs) to create a composite, ZIF-8@CNT, for the effective separation of oil/water emulsions.^73^ The oxidized CNT provided nucleation sites for the growth and deposition of ZIF-8 particles. The composite was tested for its demulsification ability on an oil/water mixture made from brine and crude oil. As shown in Figure 14, the composites had high oil removal rates over 30 min, reaching a maximum of 99.4%. The composite functions by replacing the emulsifier at the oil/water interface, bridging oil droplets to form larger ones. The mechanisms that contribute to such a high demuslification rate can be explained by the synergistic effects between ZIF-8 and the CNT structures. The imidazole ring and uncoordinated metal sites contribute to the oleophilicity of ZIF-8 and its high uptake. In addition, the surface charge on ZIF-8 caused by the positively charged metal centers can attract oil droplets via electrostatic attraction. Furthermore, the highly porous nature of the MOF allows for the adsorption of guest molecules. Highly conjugated structure of CNT facilitated π–π attraction between organic compounds. Hence, the enhanced ability to remove and separate oil from oil/water mixtures was observed as a result of the synergistic effects of the CNT and the MOF.

In another study by Han et al., a novel Zr-based MOF was deposited on polypropylene membrane to form composites with dual capability to separate oil/water mixture and function as a photocatalyst for cleaning organic pollutants.^74^ The MOF composite was developed by the in situ growth of Zr-based MOFs, namely, DUT-52 and TF-DUT-52, from 1,4-naphthalenedicarboxylic acid (H_2_NDC) and 1-trifluoroacetaminonaphthalene-3,7-dicarboxylic acid (H_2_NDC-NHCOCF_3_) as linkers, respectively. The DUT-52 and TF-DUT-52 composites showed WCAs of 132.5° and 156°, respectively. This enhanced hydrophobicity was attributed to the presence of trifluoroacetamide groups. The composite membranes showed >98% separation efficiency and a separation flux of >500 L m^–2^ h^–1^ bar^–1^ for a cyclohexane and water emulsion in addition to >85% photocatalytic degradation efficiency under ultraviolet light, hence providing the composite with the capability for self-cleaning and treatment of organic pollutants.

As mentioned earlier, one of the key elements for the effective separation of oil/water mixtures is the selective filtration capability due to the superhydrophilic or superhydrophobic nature of the materials. Hence, working in reverse, superhydrophilic composites with underwater superoleophilicity have also been studied for oil/water separation by taking advantage of the surface morphology. Obaid et al. have synthesized a superhydrophilic iron-based MOF Cr-soc-MOF-1 composite by depositing the MOF on a cellulose ester substrate to create a hydrophilic composite.^75^ The MOF composites were synthesized via vacuum-assisted self-assembly. The MOF membrane exhibited superhydrophilicity and underwater superoleophobicity, which enabled it to separate oil/water mixtures with more than 99.9% separation efficiency. Additionally, the composites showed high antifouling properties due to the synergistic effect of their surface roughness and superhydrophilicity. The composites also proved to be reusable for up to 10 filtration cycles. In another study, He et al. used the hierarchical nanostructure of CuC_2_O_4_ and HKUST-1 modified with fluoroalkylsilane for the fabrication of copper mesh membranes.^76^ The MOF composite showed a separation efficiency of 94% in 165 min owing to the synergistic effect of adsorption and photocatalytic activity. The photocatalytic mechanism was mainly attributed to the generation of superoxide and hydroxyl radicals due to the action of the MOF composite as the catalyst. In another study by Ye et al., superhydrophilic hybrid membranes of UiO-66-NH_2_ with poly vinylidenefluoride (PVDF), poly(vinyl alcohol) (PVA), and lauramidopropyl betaine (LPB) were fabricated.^77^ The composite membrane possessed excellent hydrophilicity with a WCA of 2° and showed a high water flux of 15600 L m^2–^ h^–1^. The composites also showed a high separation efficiency of 99%, in addition to the ability to filter bacteria and a self-cleaning ability.

## 5 Biodegradable MOF Composite for Oil/Water Separation

**Figure 15:**
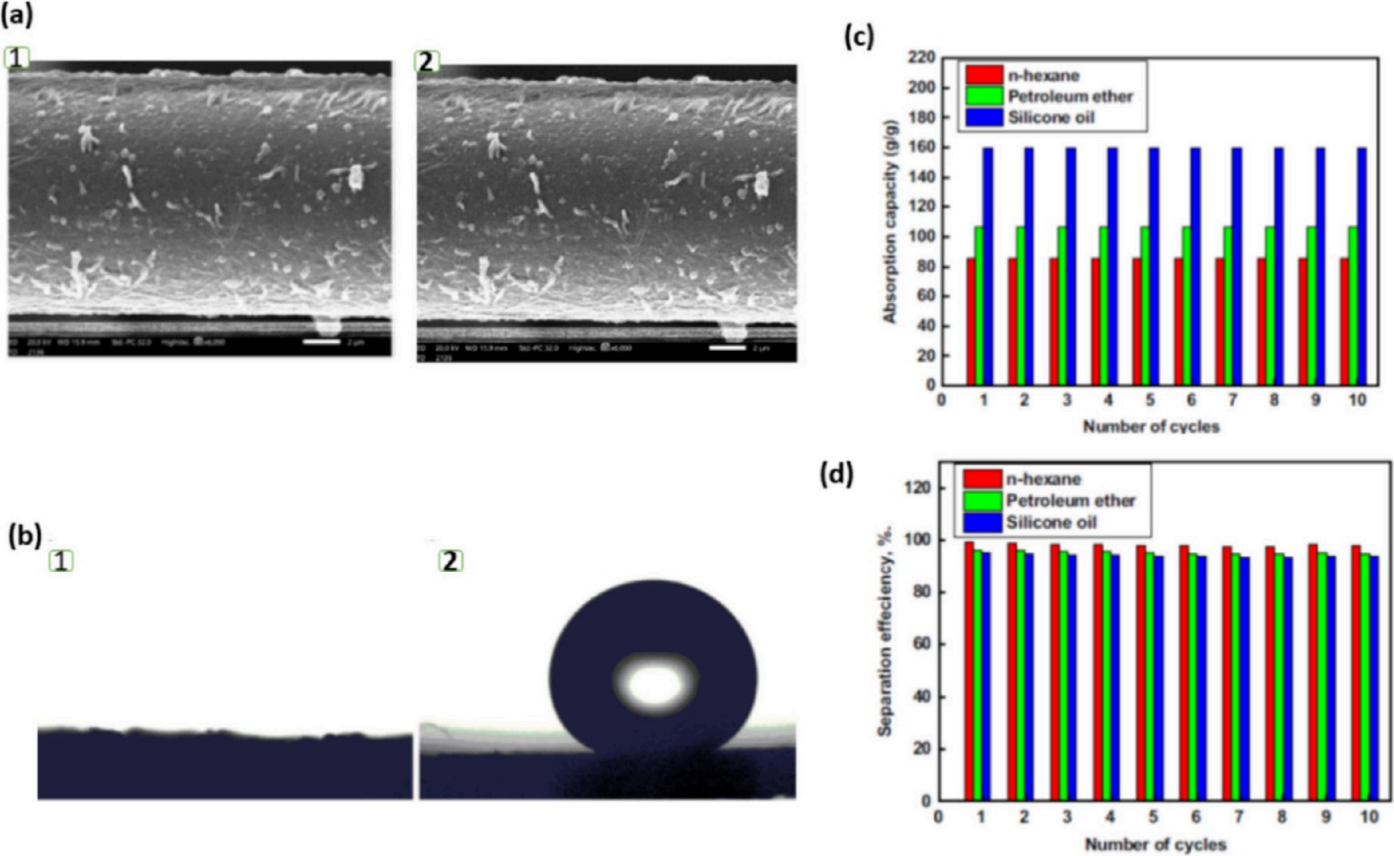
(a, b) SEM images of pristine textile fabric (1) and textile fabric modified by the Cu-Asp-MOF (2). (b) Images of a water droplet on (1) native pristine textile fabric (∼0°) and (2) textile fabric by modified by the Cu-Asp-MOF (158°) with sharply increased hydrophobicity. (c) Absorption capacity and (d) separation efficiency of the modified textile fabric for different oil/water mixtures with up to 10 separation cycles. Adapted with permission from ref (33).

The majority of organic linkers used to construct the MOFs are not of natural origin and are synthesized through multistep synthesis using raw materials derived from nonrenewable petrochemicals. Recently, the usage of naturally occurring organic linkers such as saccharides, amino acids, and nucleotides has attracted much attention for synthesis of a new class of MOFs called “Bio-MOFs”.^66^ Implementation of Bio-MOFs either as pristine materials or as composites can potentially allow a green, sustainable, and eco-friendly approach for oil/water separation. In this section, some recent examples of Bio-MOFs and their composites used in oil/water separation are discussed. Mohamed et al. reported recently a copper-based Bio-MOF, Cu-Asp-MOF, using aspartic acid as a linker prepared by an electrochemical method.^33^ The authors used stearic acid as a surfactant to reduce the surface energy of Cu-Asp-MOF. Fiber textile was then chosen as the supporting surface to fabricate a “biodegradable” MOF composite. The pristine textile showed a very smooth surface (Figure 15) with WCA = 0°, in contrast to the Cu-Asp-MOF-deposited superhydrophibic textile fiber composite, TF@Cu-Asp-MOF, with WCA = 158° ± 1°. The absorption capacities of the TF@Cu-Asp-MOF composite for *n*-hexane, petroleum ether, and silicon oil were reported as 85, 110, and 160 wt %, respectively, for 10 cycles, with a separation efficiency ranging between 95.0% and 99.4%.

More recently Li and co-workers reported the synthesis of carbon nitride–copper MOF-based composite (CN-Cu-MOF) with a WCA of 150° ± 2°, indicating the superhydrophobic nature of this MOF.^65^ In order to develop an environmentally benign composite, the group used a simple soaking method to deposit C_3_N_4_-Cu-MOF on a cotton surface. The resulting CN/Cu-MOF@cotton showed a separation efficiency of 99% for up to 10 cycles, with excellent stability in highly acidic and alkaline conditions and high absorption capacity for different oil/water mixtures, namely, castor oil, engine oil, and canola oil, without any noticeable degradation in efficiency.

Due to its appealing biodegradability, cellulose recently has been incorporated into composites with hydrophobic MOF materials for applications in oil/water separation. For example, ZIF-8 has been used to develop a cellulose acetate foam composite, ZIF-8@CA, via a thermally induced nonsolvent-induced phase separation method.^38^ This composite showed a highly hydrophobic nature, as indicated by relatively high WCA (153°) and moderate adsorption capacity range of 6.89–14.61 wt % for *n*-hexane, xylene, and soybean oil/water mixtures, and a separation efficiency of 98.7% for up to six cycles. In another study, Lu et al. fabricated cellulose fiber composites of MIL-100(Fe) with catalytic activity. The cellulose fibers were first carboxymethylated with (−COOH) groups, followed by the deposition of MIL-100(Fe) on the cotton fiber surface via in situ growth.^78^ Following this, AgCl crystals were formed on the MOF composite via the layer-by-layer method. Ag nanoparticles were formed in situ on the surface of the composite to result in a Ag@AgCl@MIL-100(Fe)/CCF composite, which exhibited hydrophilic activity and underwater oleophilicity.

## 6 Current Challenges and Future Direction

Despite the great promise of MOFs and their composites in oil/water separation, these materials should have good hydrolytic stability under both acidic and alkaline conditions for their practical applications. Although some recently developed MOFs have shown enhanced hydrolytic stability, the majority of MOFs are prone to rapid degradation in water. It must be noted that the interfacial interaction between MOFs and the supporting matrix (such as polymer, graphene oxide, etc.) plays a very important role in the stability of the resulting composites and, therefore, should be studied in detail. For example, a reverse approach for developing a hydrophilic MOF–polymer composite has been studied recently.^77^ Other great challenges associated with water/oil separation are linked to the type of separation materials (adsorbent or membrane) used, namely, transferring and processing of the bulk material after each separation cycle,^86^ recyclability, scalability, and cost. When considering materials for large-scale oil/water separation, factors related to recyclability and sustainability are of great concern. Currently, the development of recyclable MOFs, Bio-MOFs, and biodegradable MOFs has attracted much attention and is being considered as the future direction of MOF-based materials for oil/water separation.^87^ Implementation of MOFs in bulk water treatment is still unexplored, and for large-scale applications MOFs need to be manufactured from cost-effective as well as renewable feedstocks and resources. Being a relatively new area, the cost of the scalable MOF composites has yet to be explored. Sustainability and recyclability are going to be more crucial for future applications, and the lifecycle assessment and circularity of the materials will need to be studied in more detail for practical implementation.^88^ Being a relatively new area, there is also a lack of consistency in reporting the efficiency of the materials. For example, permeation data provides a better way to compare effectiveness compared to flux, and more coherence in the reported results will help to address this issue.^36^ The emerging trend of using machine learning to identify promising MOFs (as being applied in other areas, such as hydrogen storage) is also expected to contribute to the development of more efficient MOF–polymer composites for oil/water separation.^89,90^

## 7 Conclusion

Purification of water contaminated with oil can aid in the remediation of water and can help to prevent ecological damage from oil spills and urban and industrial pollution. Recently, various materials and methods have been studied for this important application due to their intriguing properties, including ultrahigh surface areas, tunable pore geometries and chemical properties, and surface adaptability, with numerous studies exploring the development of hydrophobic MOFs for selective adsorption of the oil phase. In this Review, the recent developments and main strategies of using MOFs and their composites in oil/water separation have been discussed. Common approaches involve the use of aromatic, fluorine, or long-chain alkyl-functionalized linkers as building blocks for the synthesis of hydrophobic MOFs. The symmetry of the linker, as well as its coordination with the metal clusters, also contributes to surface morphologies and surface energies that may induce hydrophobicity. Other strategies involve the post-synthetic modification of the MOFs by the addition of hydrophobic functional groups containing fluorine and long-chain alkyl groups either on the linker or as capping ligands on the coordinatively unsaturated metal ions. Additionally, the recent focus on composites has shown that the MOFs can be incorporated into a composite material for the enhancement of certain features like stability, hydrophobicity, and processability and may also provide a synergistic effect on the enhancement of hydrophobicity. Several materials for the fabrication of composites have been used, including graphene oxide, metal meshes, and melamine sponges. More recently, biodegradable polymers, such as cellulose and polylactic acid, have been investigated for more environmentally friendly MOF–polymer composites for oil/water separation. With the rising number of studies, the importance of this class of materials is evident, and it can be assumed that the current concern over environmental pollution caused by direct and indirect oil spillage deserves more material-based approaches to find solutions for this critical problem, which is directly linked to “access of clean water” and “life below water”, two of the Sustainable Development Goals (SDGs) set by the United Nations.
